# Supplementary material for: Slab control on the mega-sized North Pacific ultra-low velocity zone
Source: Nat Commun. 2022 Feb 24;13:1042. doi: 10.1038/s41467-022-28708-8 (PMC8873298; doi:10.1038/s41467-022-28708-8)
Supplement: Supplementary file 1 — Supplementary Information [file 41467_2022_28708_MOESM1_ESM.pdf]

Supplementary Information for "Slab Control on the Mega-sized North Pacific Ultra-low Velocity Zone"

Jiewen Li, Daoyuan Sun, Dan J. Bower

Contents

Supplementary Note 1. Sensitivity tests for ULVZ models 2
Note 1.1 2D sensitivity tests along the N-S profile 2
Note 1.2 3D sensitivity tests from two orthogonal profiles 3
Note 1.2.1 δV_S and H 4
Note 1.2.2 Southern boundary 5
Note 1.2.3 Northern boundary 5
Note 1.2.4 Western boundary 5
Note 1.2.5 Shape 6
Note 1.2.6 Density 7
Note 1.2.7 Non-uniform δV_S 7
Note 1.2.8 CMB topography 10
Supplementary Note 2. Structure of the northern edge of the Pacific LLVP in previous studies 11
Supplementary Note 3. D″ discontinuity at the northern and northeastern edge of the Pacific LLVP 11
Supplementary Note 4. Deep-rooted Hawaiian plume 12
Supplementary References 14

Supplementary Note 1. Sensitivity tests for ULVZ models

We generate all synthetics in a broad frequency band. In 2D simulations, we select the filter band from 5–50 s for events A–D, which allows a better resolution of the ULVZ structure. For 3D simulations, due to the heavy computation, the shortest period of event B is at ~8 s, so we filter data from 10–50 s. For event F, we also filter data from 10–20 s, which is used in Cottaar and Romanowicz (2012), to enable a better comparison.

Here we perform a series of sensitivity tests on the parameters of the ULVZ, i.e., shear velocity perturbation (δV_S), height (H), size, shape, density, and possible existence of heterogeneities. A summary of the sensitivity tests for the ULVZ models is listed in Supplementary Table S3.

Note 1.1 2D sensitivity tests along the N-S profile

Along the N-S profile, at the period of 5–50 s, the waveforms are similar for box-car and dome-like ULVZ shapes (Supplementary Figure 8). Thus, for simplicity, we examine the trade-offs between δV_S and H using a 2D box-car shape ULVZ model extending from 34° to 46° (taking event B as the reference at 0°) (Supplementary Figure 9a). We vary δV_S (-30%–0) relative to IASP91[1] and H (0–300 km) with an increment of 2% and 10 km, respectively. This determines the best 2D ULVZ model with δV_S of ~ -10% and H of 50 km (Supplementary Figure 9a) based on the maximum cross-correlation coefficient (CC) of events A–D. This optimal model predicts S and ScS waveforms for all events as well as sS and sScS for events A and B (Supplementary

Figure 9b). For models with stronger δV_S and lower H, the synthetics have strong arrivals after ScS, which are not observed in the data, even though the CC is high. In addition, we explore several 2D ULVZ models with different extents to show the influence of varying the southern (Supplementary Figure 10a-b) and northern boundaries of the ULVZ (Supplementary Figure 10c-d). If the southern boundary extends further to the south (Supplementary Figure 10a) or the northern boundary extends further to the north (Supplementary Figure 10c) relative to our preferred ULVZ model in Supplementary Figure 9, the synthetics have more delayed ScS or sScS compared to data. Conversely, if the southern boundary moves further to the north (Supplementary Figure 10b) or the northern boundary moves further to the south (Supplementary Figure 10d), the synthetics have less delayed ScS or sScS compared to data, resulting in a low CC. Overall, the optimal ULVZ model (δV_S = -10%, H = 50 km) in Supplementary Figure 9 provide the maximum CC and best waveform fits, demonstrating that the southern and northern boundaries can be approximately determined with these 2D sensitivity tests.

Note 1.2 3D sensitivity tests from two orthogonal profiles

We study the trades-offs in 3D models by fitting waveforms of event B on the N-S profile and event F on the W-E profile. For event B, we divide the data into 3 profiles: Profile NS-I, a distance profile at the azimuth of 0°–10° (e.g. Fig. 5a); Profile NS-II, a distance profile at the azimuth of 10°–30° (e.g. Supplementary Figure 6a); Profile NS-III, an azimuthal profile with distance (Δ) of 90°–100° (e.g. Supplementary Figure 6b).

For event F, we select an azimuthal profile WE with Δ of 100°–110° (e.g. Fig. 5b) as used in Cottaar and Romanowicz (2012). For event F, we select a time window containing Sdiff and its postcursor to calculate CC. The eastern boundary of the ULVZ can be defined at which S and ScS merge along the azimuthal profile NS-III at an azimuth of ~25° (Supplementary Figure 6b), so we alter δV_S, H, boundary (southern, northern, western) positions, shape, density, and non-uniformity variations based on our best ULVZ model and examine how these parameters affect the goodness of waveform fitting.

Note 1.2.1 δV_S and H

We examine the trade-offs between δV_S and H of ULVZ models with the shape fixed as the NP ULVZ model (Supplementary Figures 12-14). We vary δV_S (-30%–0) and H (0–100 km) by increments of 5% and 20 km, respectively. Supplementary Figures 12-14 shows that the CC are the highest (equivalently, σ_{L1} or σ_{L2} are the lowest) when δV_S and the height are close to the NP ULVZ model (δV_S = -10%, height = 50 km) for both events B and F. A high CC (or low σ_{L1} /σ_{L2}) can also be achieved for both events B and F with δV_S of -15% and H of 20 km. However, with stronger δV_S and lower H (δV_S = -15%, H = 20 km), the synthetics have delayed ScS and strong arrivals after ScS on the profile NS-II (marked with a red dashed line in Supplementary Figure 15A), generated by multiple reflection inside of the ULVZ owing to the large δV_S. For a more extreme model (δV_S = -40%, H = 10 km) as in Supplementary Figure 15B, the Sdiff postcursors are too strong with a long coda wavetrain on the profile WE. With weaker

δV_S (e.g. -5%), the model is not able to produce strong postcursors after Sdiff as observed in data on the profile WE, no matter how we vary H. Hence, a moderate δV_S of -10% is preferred to give a high CC and reproduce the waveforms for both events B and F.

Note 1.2.2 Southern boundary

When the southern boundary of the western side of the NP ULVZ model is displaced further to the north (Supplementary Figure 15C), the ScS delays at large distance are not sufficient on the profile NS-I. Also, the CC is much lower because the Sdiff postcursors arrive much earlier compared to data on the profile WE. When the southern boundary is located further to the south (Supplementary Figure 15D), the amplitudes of ScS of the profile NS-I are much weaker at large distance, although there is little effect on the profile WE.

Note 1.2.3 Northern boundary

When the northern boundary of the western side of the NP ULVZ is displaced further to the south (Supplementary Figure 15E), the ScS delays at large distance on the profile NS-I are not sufficient and the Sdiff postcursors arrive much earlier compared to data on the profile WE.

Note 1.2.4 Western boundary

When we shrink the western boundary by moving it to the east (Supplementary Figure 15F), it has little influence on the waveforms observed for event B. However,

on the profile WE, the Sdiff postcursors arrive much earlier and the amplitudes are small at the azimuth of 48°-55° compared to data. When we increase H to 80 km with δV_S of -10% (Supplementary Figure 15G), synthetics match data well on the profile WE, but ScS has more delays at large distance on the profiles NS-I, II, III compared to data. It is difficult to constrain the western boundary of the ULVZ precisely because of the lack of data with azimuth smaller than 4° (Supplementary Figure 6b). However, the separations between Sdiff and postcursors on the profile WE are sensitive to the ULVZ extent (Supplementary Figure 15F-G). Thus, it may provide extra constraints on the lateral extent of the western boundary of the NP ULVZ for a given δV_S.

Note 1.2.5 Shape

Here, we show three ULVZ models with different shapes. Our 3D ULVZ model inherits the HM ULVZ in the east, but extends more to the west. Supplementary Figure 15H shows a model at the same location as the HM ULVZ but its δV_S and H are the same as our best model (δV_S = -10%, H = 50 km). The ScS delays are not sufficient at large distance on the profile NS-I compared to data, so the extra western side in NP ULVZ is necessary to match the delayed ScS. Also, the Sdiff postcursors arrive much earlier compared to the data of the profile WE. If the ULVZ model has a trapezoid shape (Supplementary Figure 15I), the predicted ScS arrivals are delayed more with distance larger than 95° on the profile NS-II. Furthermore, the Sdiff postcursors on the profile WE are strong at small azimuth even down to 40°, which is not compatible with data. If the ULVZ model has an irregular shape (Supplementary Figure 15J), the Sdiff

postcursors on the profile WE are also strong at small azimuth even down to 40°. In contrast, the NP ULVZ with local geometric curvature in the east is able to fit data with respect to move-outs and amplitude variation between Sdiff and its postcursors along the azimuth (Fig. 5b).

Note 1.2.6 Density

In Supplementary Figure 15K-L, ULVZ models with a density perturbation (±10%) predict almost identical waveforms to the NP ULVZ model which has no density perturbation. In addition, a ULVZ model with a density increase of 20% (Supplementary Figure 15M) predicts slight waveform amplitude variation. Hence, the seismic phases at the period in this study (>10 s) have negligible sensitivity to the density of the ULVZ.

Note 1.2.7 Non-uniform δV_S

At some length scale, potentially below the seismic detection threshold, a ULVZ may be heterogeneous. Thus, we test several non-uniform ULVZ models with different δV_S or H at different parts of the ULVZ.

As displayed in Supplementary Figure 15N-P, we partition the NP ULVZ model into two parts. The δV_S and H of part 1 are the same as the NP ULVZ (δV_S = -10%, H = 50 km) while part 2 has the same δV_S and height as the HM ULVZ (δV_S = -20%, H = 20 km). In Supplementary Figure 15N, the overall shape of ULVZ is still the same as the NP ULVZ model, but ScS arrivals are delayed much more than the data and strong extra arrivals between ScS and sS are evident on the profile NS-II. Such a model also

generates much stronger Sdiff postcursors with long codas on the profile WE. In Supplementary Figure 15O, we separate part 1 and part 2 by a distance such that they are distinct (unconnected) structures. This model fails to explain ScS on the profile NS-III. In particular, the ScS delays are much more at large azimuth even up to 27°. In Supplementary Figure 15P, we move part 1 further to the west. This model fails to explain the delayed ScS at large distance on the profile NS-I. It also generates Sdiff postcursors at small azimuth. In addition, we change δV_S and H of part 2 to be the same as part 1 (δV_S = -10%, H = 50 km, Supplementary Figure 15Q). Also, this model fails to explain the ScS on the profile NS-III because the ScS delays are much more at large azimuth even up to 22°.

Supplementary Figure 15R shows a model with gradual variation of δV_S in depth, in which the δV_S varies from 0 at the top of the ULVZ to -30% at the CMB. Owing to the strong δV_S near the CMB, ScS arrivals are delayed much more than the data and strong extra arrivals between ScS and sS are developed on the profile NS-II. Such a model also generates much stronger Sdiff postcursors with long codas on the profile WE. In Supplementary Figure 15S, a model with δV_S varying from -5% at the top of the ULVZ to -15% at the CMB can fit data both for events B and F almost as good as for our best uniform ULVZ model (δV_S = -10%, H = 50 km). Supplementary Figure 15R-S suggests that a radial decrease of δV_S by about 10% from the top to the bottom of the NP ULVZ can predict data well. However, the radial decrease of δV_S in the NP ULVZ should not exceed 20%.

In Supplementary Figure 15T, δV_S varies randomly from -5% to -15% at a short-

wavelength scale with a correlation length of ~60 km laterally. This model can also fit data for both events B and F almost as good as for our best uniform ULVZ model (δV_S = -10%, H = 50 km). Additionally, to explore possible lateral heterogeneities in the NP ULVZ, we generate several pseudo-tomographic models. Firstly, we enhance S40RTS[2] (Supplementary Figure 15U) and SEMUCB-WM1[3] (Supplementary Figure 15W), where the δV_S in the range of the NP ULVZ is amplified to have a mean δV_S of -10% and H of 50 km. The enhanced S40RTS model in Supplementary Figure 15U has a relatively weak heterogeneities, with δV_S varying from -9% to -11%, and it can predict data well. However, if we change the mean δV_S to -15% (Supplementary Figure 15V), the synthetics have delayed ScS on the N-S profile and much stronger Sdiff postcursors. By contrast, the enhanced SEMUCB-WM1 model (Supplementary Figure 15W) has δV_S varying from -8% and -12% at a wavelength of ~500 km. Although this model can predict the Sdiff postcursors, it fails to match the data on the N-S profile (Supplementary Figure 15W). Secondly, we attribute the measured $\delta t_{ScS\text{-}S}$ along the N-S profile to lateral variations of the NP ULVZ. Two heterogeneous ULVZ models are generated by fixing H of 50 km (Supplementary Figure 15X) or δV_S of -10% (Supplementary Figure 15Y), and interpolating in the whole range of the NP ULVZ according to the measured $\delta t_{ScS\text{-}S}$. In Supplementary Figure 15X, the δV_S varies from -12% to -8% at a short wavelength of ~200 km. In Supplementary Figure 15Y, the H varies from 40 to 60 km. Both models provide an overall good fit. Furthermore, we also test a model consisting of many small disconnected patches of ULVZs with a strong δV_S of -40% and H of 20 km (Supplementary Figure 15Z), this model cannot produce

appropriate ScS delays and strong Sdiff postcursors as observed in data. Supplementary Figure 15U-Y implies that the lateral δV_S variation in the NP ULVZ is not likely to exceed to 4% at a wavelength of ~200 km, which is compatible with a large Fresnel Zone of Sdiff for 10 s period (Supplementary Figure 15A). Hence we determine that the NP ULVZ must be a continuous block at large scale, rather than many small disconnected patches. However, small and strong variations might exist, which is indistinguishable at the period (> 10 s) in this study.

In summary, although a heterogeneous ULVZ cannot be ruled out, our uniform NP ULVZ model (δV_S = -10%, H = 50 km) as a whole explains the waveform data at the period in this study. Thus, our NP ULVZ model represents well the bulk properties, both geometry and velocity, of the ULVZ located at the northern boundary of the Pacific LLVP.

Note 1.2.8 CMB topography

We examine how CMB topography affects ScS waveforms along the N-S profile at the period of 5–50 s and 10–50 s used in this study (Supplementary Figure 16). Synthetics are generated by the 2D finite difference code. For the two models in Supplementary Figure 16, the CMB with topography has a width (L) of 15°, comparable to the size of the NP ULVZ, and a relief of 20 km. Synthetics show a very small increase of the ScS amplitude for a concave CMB topography (Supplementary Figure 16c) and opposite for the convex one (Supplementary Figure 16d). These calculations suggest that the CMB topography has little effect on waveforms in this study.

Supplementary Note 2. Structure of the northern edge of the Pacific LLVP in previous studies

A few studies have demonstrated the complicated northern boundary of the Pacific LLVP. He and Wen[4] outline the boundary of the Pacific LLVP using the differential travel time between S and ScS, SS and ScSScS (ScS2). They determine the height of the LLVP up to ~500 km because it shows the strongest correlations between δt_{ScS-S} and δt_{ScS} if using this cut-off depth to correct travel times for δt_{ScS-S} and δt_{ScS}. However, in our study we observe a continuous change of S-wave travel time residuals from more event-receiver pairs by taking advantage of the deployment of USArray in Alaska. It is significant to identify the sharp discontinuity (-2% in δV_S) on top of the LLVP, because it suggests a compositionally distinct LLVP (relative to ambient mantle) instead of a purely thermal contrast that would result in a smooth change in velocity perturbation at its top. Furthermore, Frost and Rost[5] also conclude that the northern boundary of the Pacific LLVP has a shallow dip angle. With limited measurements, however, it is not surprising to see differences among the obtained dip directions of the LLVP edge.

Supplementary Note 3. D″ discontinuity at the northern and northeastern edge of the Pacific LLVP

In the GyPSuM tomographic model[6], two distinct high S-wave velocity regions at the CMB are situated at the northern and northeastern edge of the Pacific LLVP (Fig. 1b). For the northern part, its existence can be verified by the observed diminishing S-wave travel time residuals at the large distance of events A–D (Fig. 3c). He and Wen[4]

also declare that a D″ discontinuity is next to the LLVP further to the north. This discontinuity is 220 km above the CMB and has a +2% jump in shear velocity. Geographically, the NP ULVZ (presented in this work) is situated between the LLVP and the high velocity region, and partially penetrates into the LLVP. Along the northeastern edge of the Pacific LLVP, there are many proposed ULVZs and also a high velocity region located further east (Fig. 1b). This high velocity region is also supported by studies from Breger and Romanowicz[7] and Sun et al.[8] These high velocity regions are generally thought to be slab debris originating from past subduction in the northern Pacific.

Supplementary Note 4. Deep-rooted Hawaiian plume

We plot depth cross-sections of tomography models (Supplementary Figure 18): GyPSuM[6] (S), S20RTS[9] (S), GLAD-M25[10] (S), SEMUCB-WM1[3] (S) and DETOX-P2[11] (P). Some models (such as SEMUCB-WM1) exhibit a concentric conduit arising from the lowermost mantle, forming a plume beneath Hawaii, with a northward tilting feature caused by southward mantle flow in the lower mantle. Combined with the thinning transition zone identified beneath the south-southeast of Hawaii[12,13], the Hawaiian plume is more likely to be rooted at the northeastern edge of the Pacific LLVP instead of above the NP ULVZ.

Also, there are several deep-rooted plumes showing bilateral zonation with enriched and depleted trends oriented towards and away from LLVP interiors[14,15] (Supplementary Figure 19). This indicates that the materials of deep-rooted plumes

with bilateral zonation may originate from different reservoirs.

Supplementary References

1 Kennett, B. L. N. & Engdahl, E. R. Traveltimes for Global Earthquake Location and Phase Identification. *Geophys J Int* **105**, 429-465 (1991).

2 Ritsema, J., Deuss, A., van Heijst, H. J. & Woodhouse, J. H. S40RTS: a degree-40 shear-velocity model for the mantle from new Rayleigh wave dispersion, teleseismic traveltime and normal-mode splitting function measurements. *Geophys J Int* **184**, 1223-1236 (2011).

3 French, S. W. & Romanowicz, B. Broad plumes rooted at the base of the Earth'smantle beneath major hotspots. *Nature* **525**, 95-99 (2015).

4 He, Y. M. & Wen, L. X. Geographic boundary of the "Pacific Anomaly" and its geometry and transitional structure in the north. *J Geophys Res-Sol Ea* **117** (2012).

5 Frost, D. A. & Rost, S. The P-wave boundary of the Large-Low Shear Velocity Province beneath the Pacific. *Earth Planet Sc Lett* **403**, 380-392 (2014).

6 Simmons, N. A., Forte, A. M., Boschi, L. & Grand, S. P. GyPSuM: A joint tomographic model of mantle density and seismic wave speeds. *J Geophys Res-Sol Ea* **115** (2010).

7 Breger, L. & Romanowicz, B. Three-dimensional structure at the base of the mantle beneath the central Pacific. *Science* **282**, 718-720 (1998).

8 Sun, D. *et al.* Slab Control on the Northeastern Edge of the Mid-Pacific LLSVP Near Hawaii. *Geophys Res Lett* **46**, 3142-3152 (2019).

9 Ritsema, J., van Heijst, H. J. & Woodhouse, J. H. Complex shear wave velocity structure imaged beneath Africa and Iceland. *Science* **286**, 1925-1928 (1999).

10 Lei, W. J. *et al.* Global adjoint tomography-model GLAD-M25. *Geophys J Int* **223**, 1-21 (2020).

11 Hosseini, K. *et al.* Global mantle structure from multifrequency tomography using P, PP and P-diffracted waves. *Geophysical Journal International* **220**, 96-141 (2020).

12 Kemp, M., Jenkins, J., Maclennan, J. & Cottaar, S. X-discontinuity and transition zone structure beneath Hawaii suggests a heterogeneous plume. *Earth Planet Sc Lett* **527** (2019).

13 Schmerr, N., Garnero, E. & McNamara, A. Deep mantle plumes and convective upwelling beneath the Pacific Ocean. *Earth Planet Sc Lett* **294**, 143-151 (2010).

14 Heyn, B. H., Conrad, C. P. & Tronnes, R. G. Core-mantle boundary topography and its relation to the viscosity structure of the lowermost mantle. *Earth. Planet. Sci. Lett.* **543** (2020).

15 Torsvik, T. H., Steinberger, B., Ashwal, L. D., Doubrovine, P. V. & Tronnes, R. G. Earth evolution and dynamics-a tribute to Kevin Burke. *Can J Earth Sci* **53**, 1073-1087 (2016).

16 Cottaar, S. & Romanowicz, B. An unsually large ULVZ at the base of the mantle near Hawaii. *Earth Planet Sc Lett* **355**, 213-222 (2012).

17 Megnin, C. & Romanowicz, B. The three-dimensional shear velocity structure of the mantle from the inversion of body, surface and higher-mode waveforms. *Geophys J Int* **143**, 709-728 (2000).

18 Hassan, R., Muller, R. D., Gurnis, M., Williams, S. E. & Flament, N. A rapid burst in hotspot motion through the interaction of tectonics and deep mantle flow. *Nature* **533**, 239-242

(2016).

Supplementary Figures 1-19 and Supplementary Tables 1-3

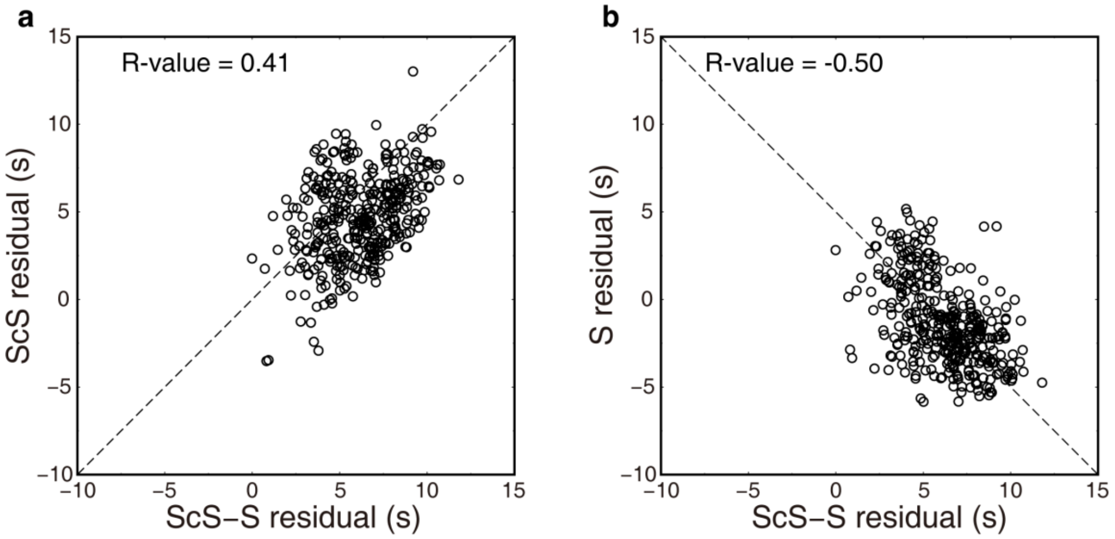

Supplementary Figure 1: Relationship between observed S/ScS travel time residuals ($\delta t_S/\delta t_{ScS}$) and differential travel time residuals between S and ScS (δt_{ScS-S}) after correction for IASP91[1]**.** The dashed lines have a slope of 1 for left panel and -1 for right panel. Travel time residuals are corrected to one common station (UNV) to remove possible errors of event origin time and location. Pearson's R-values quantify the correlations.

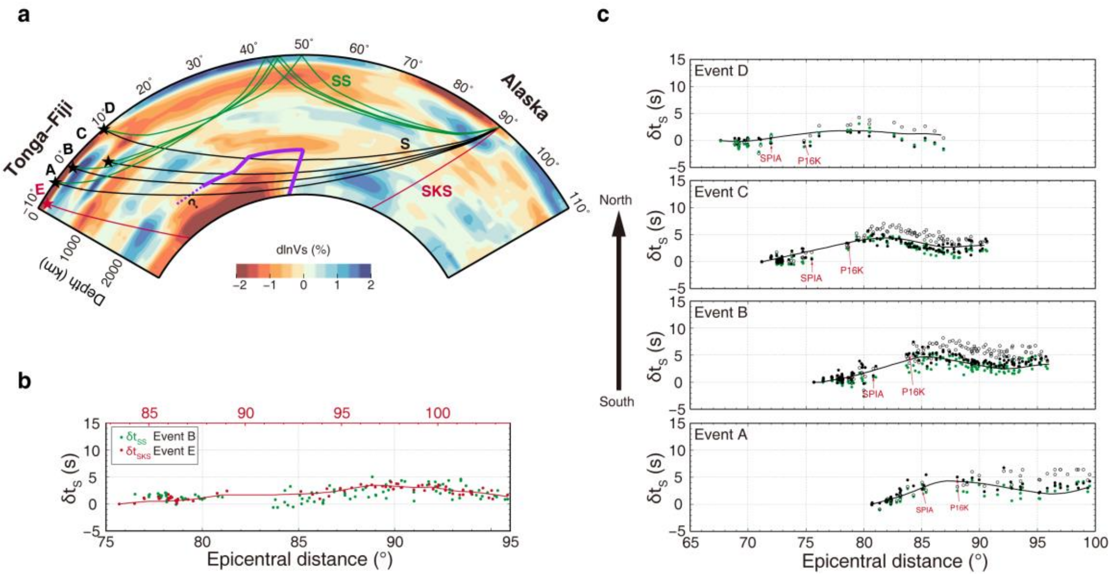

Supplementary Figure 2: Correction for S-wave travel time residuals. a, Depth cross-section from Tonga-Fiji to Alaska in GyPSuM[6]. The ray paths of S (black), SKS (red) and SS (green) are plotted at one common station from different events (stars). The purple line outlines our inferred LLVP while the southernmost portion (purple dashed line) is not well constrained. **b,** SKS travel time residuals of event E (red) corresponding to upper axis and SS travel time residuals of event B (green) corresponding to lower axis relative to IASP91[1]. **c,** S travel time residuals (hollow circles) relative to IASP91[1] and after correction (black solid circles) using SKS travel time residuals and after correction (green solid circles) using SS travel time residuals in **b** along with predictions from the new LLVP model (solid line) in **a**. Events are arranged from south to north. Two common stations (SPIA and P16K) show different S travel time trends of events A–D. Data are selected in an azimuth range of 0°–10°. Note similar trends but with a minor magnitude difference of δt_S after correction using SKS or SS of events A–D.

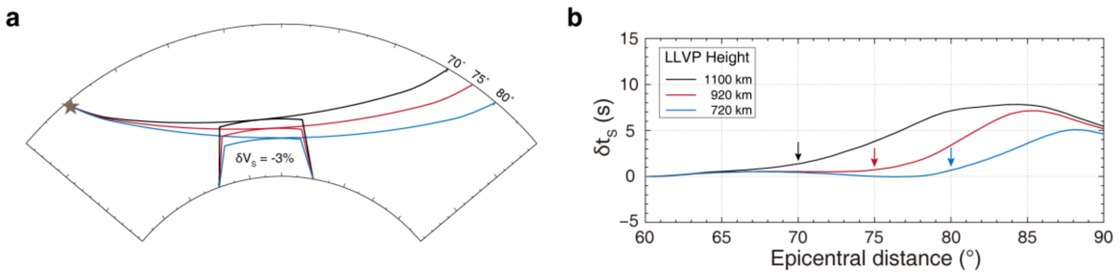

Supplementary Figure 3: Relation between the distance of the initial increase of the δt_S and the LLVP height. a, Depth cross-section shows the LLVP structure with different heights (black for 1100 km, red for 920 km, blue for 720 km) and the corresponding rays of S-wave turning at the top of the LLVP. **b,** S-wave travel time anomalies for different LLVP structures presented in **a.** Note the distances of the initial increase of the δt_S (marked by arrows) correspond to S starting to sample the LLVP.

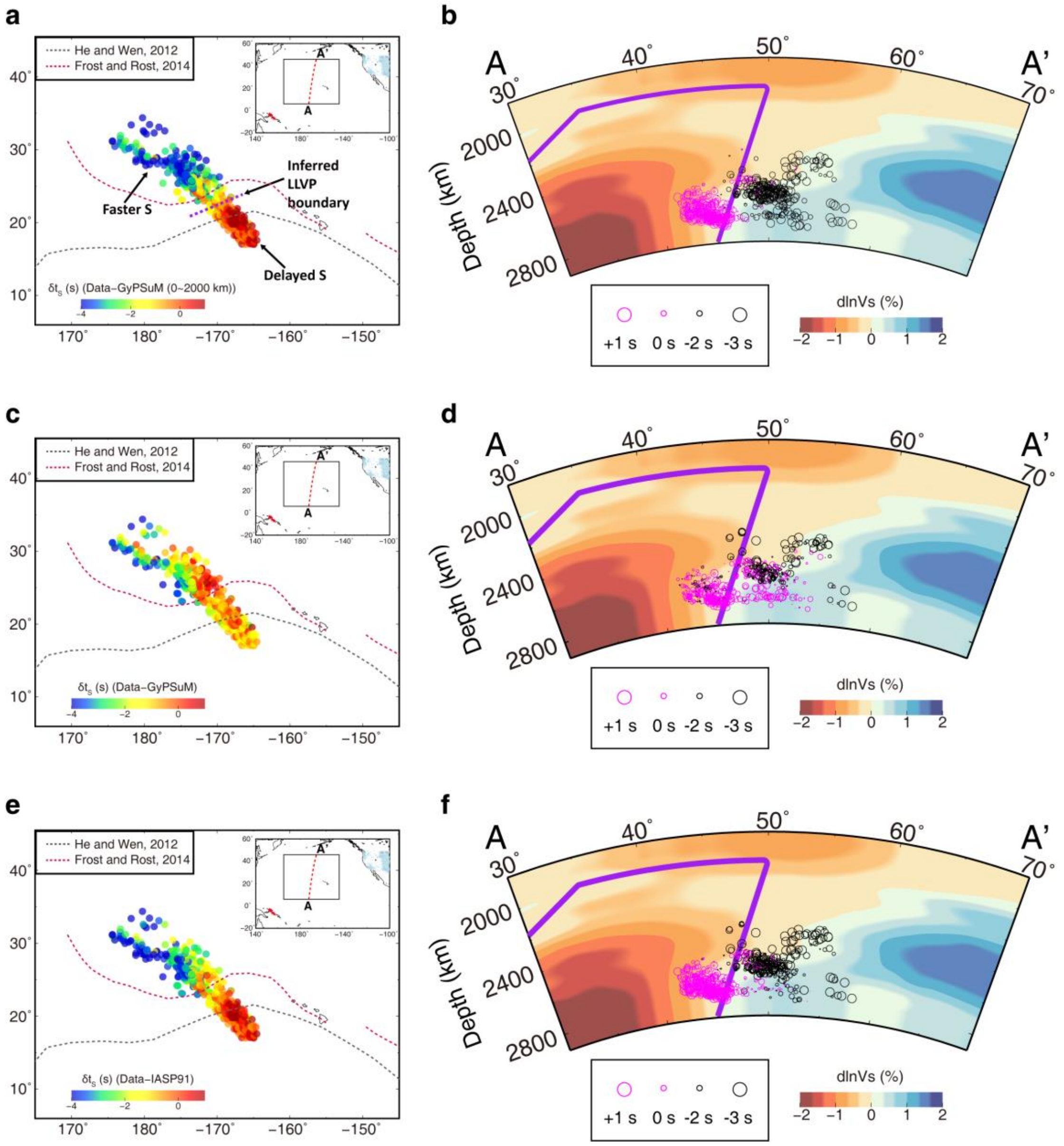

Supplementary Figure 4: S-wave travel time residuals. a, Enlarged view marked with black box in inset showing S travel time residuals for events in Solomon Islands recorded in North America on the west-east profile after correction to a common station MLAC relative to GyPSuM[6] at a depth of 0–2000 km. Results are plotted at their turning points. Note the sudden change of travel time residuals across the inferred northern boundary of the Pacific LLVP (purple dashed line). The grey and red dashed lines are LLVP boundaries from He and Wen[4] and Frost and Rost[5], respectively. b**,** Depth cross-section from GyPSuM along AA' (see inset in **a)** with S travel time residuals in **a** projected as color coded circles. The purple line outlines our inferred LLVP surface by separating positive (magenta) and negative (black) travel time residuals. **c-d,** Same as **a-b** but relative to GyPSuM. **e-f,** Same as **a-b** but relative to IASP91[1].

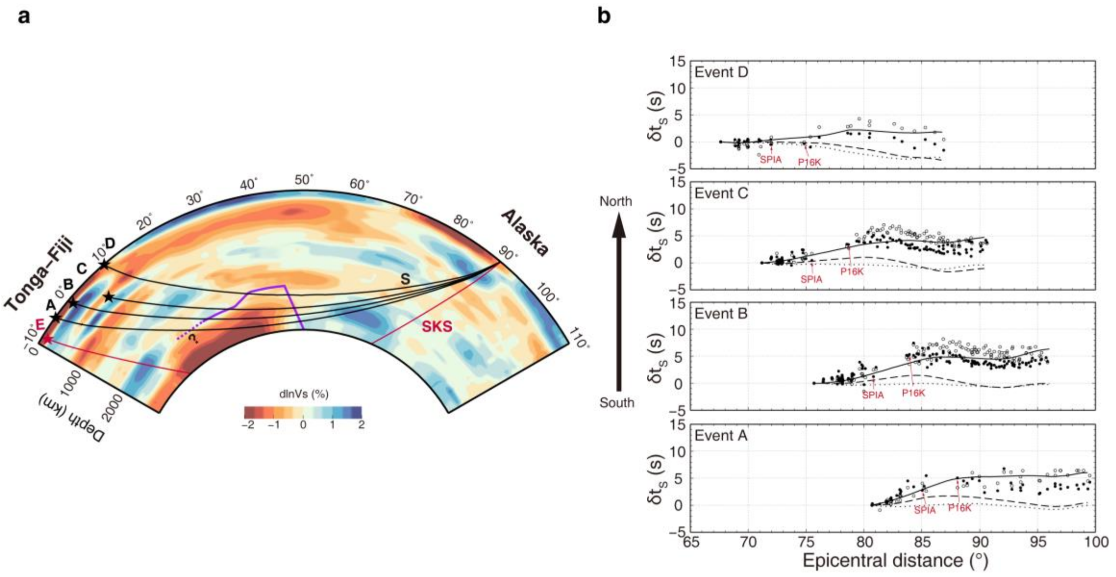

Supplementary Figure 5: Comparison of S-wave travel time residual predictions with data. a, Depth cross section from Tonga-Fiji to Alaska in GyPSuM[6]. The ray paths of S (black) and SKS (red) are plotted at one common station from different events (stars). The purple line outlines an alternative LLVP with a south dipping and a wider bottom structure, while the southernmost portion (purple dashed line) is not well resolved. **b,** S-wave travel time residuals (hollow circles) relative to IASP91[1] and after correction (solid circles) using SKS travel time residuals, along with predictions from GyPSuM (dashed line), GyPSuM at a depth of 0–2000 km (dotted line) and LLVP model (solid line) in **a**. Events are arranged from south to north. Two common stations (SPIA and P16K) show different S travel time trends of events A–D. Data are selected in an azimuth range of 0°–10°.

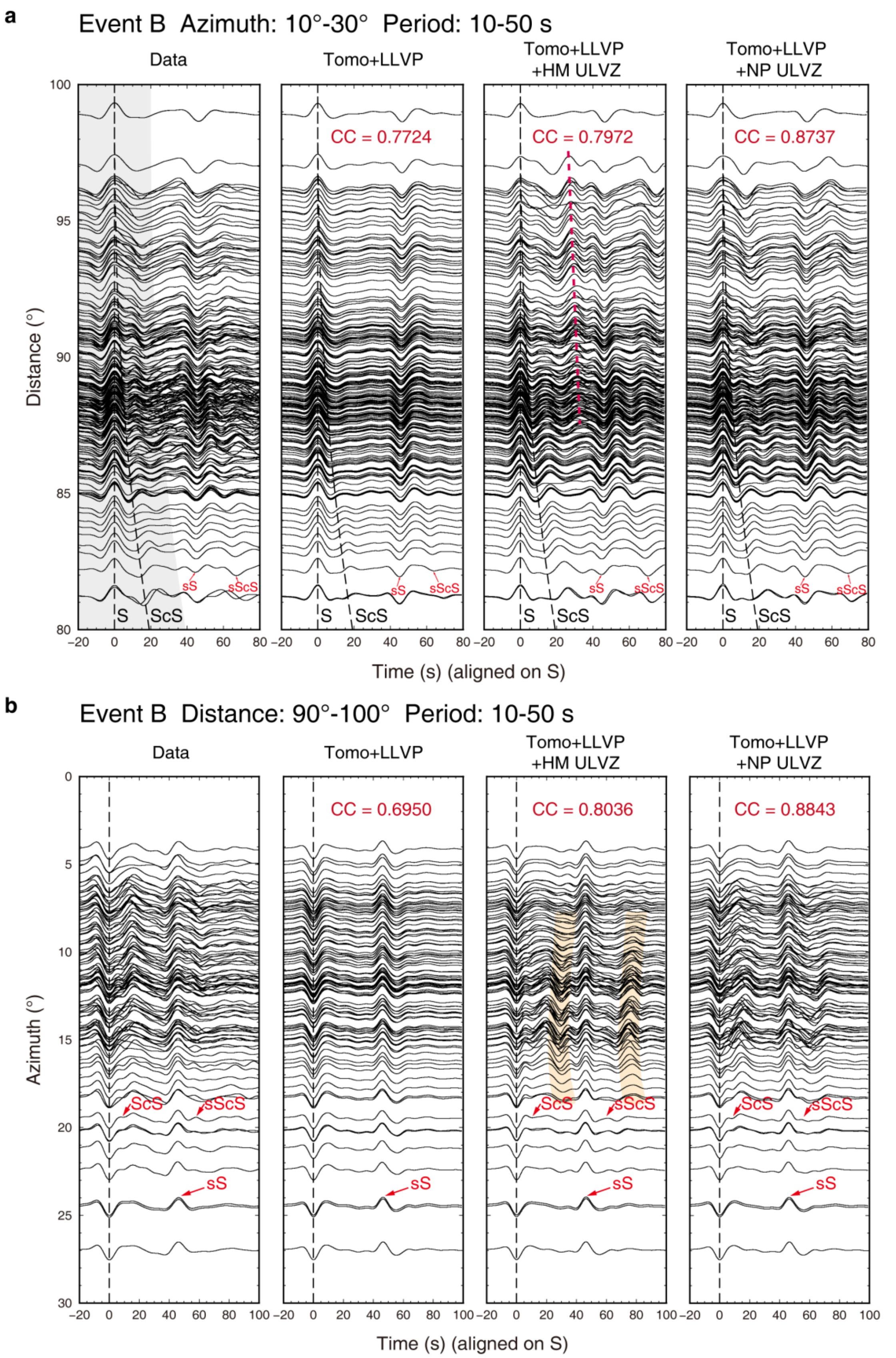

Supplementary Figure 6: Comparison between the data and 3D synthetics. a, Tangential components of event B in 10°–30° azimuth range are aligned on S and are

bandpass filtered from 10–50 s. The dashed lines in each panel show the S and ScS arrival times predicted by IASP91[1]. The depth phases sS and sScS are also labeled. The red dashed line indicates the strong postcursors after ScS (not seen in the data) caused by the large velocity reduction in the HM ULVZ. Average cross-correlation coefficients (CC) between data and synthetics are calculated in the time window indicated by the grey shaded region. **b,** Tangential components of event B in 90°−100° distance range. The differential travel time between S and ScS is mainly caused by the ULVZ as ray paths of S and ScS are extremely close to each other in this distance range. Tangential components are aligned on S and are bandpass filtered from 10–50 s. The depth phase sS is also labeled. The yellow patches denote the strong postcursors after S and sS arrivals. Columns in **a** and **b** from left to right are data, our inferred LLVP model, LLVP model plus HM ULVZ[16], LLVP model plus NP ULVZ, respectively. Average cross-correlation coefficients (CC) and misfits between data and synthetics are calculated in the grey shaded region indicated in **a**.

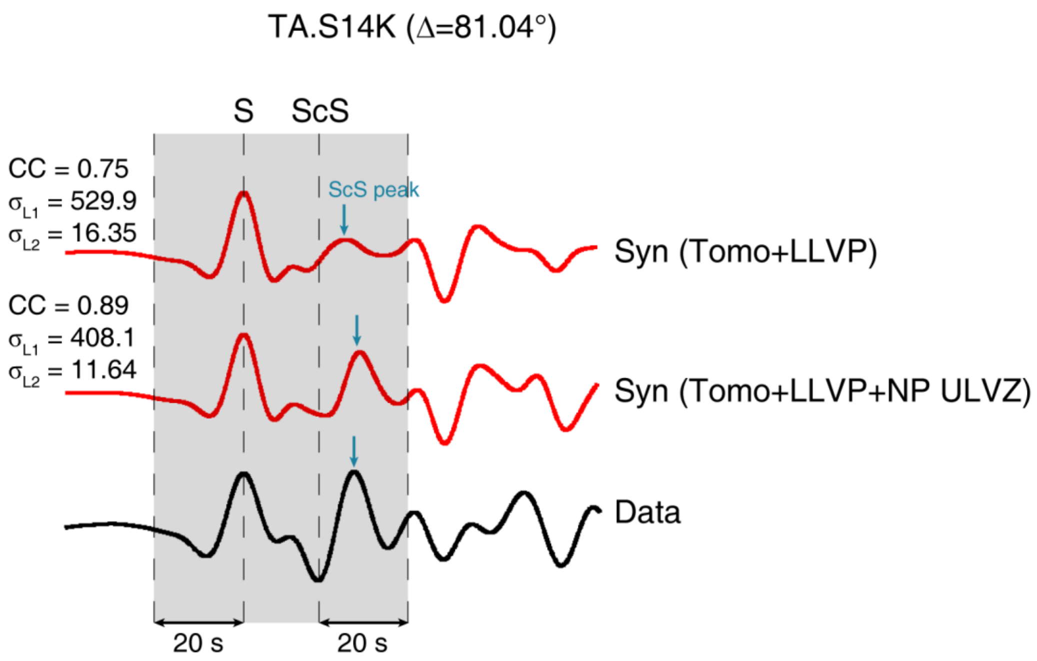

Supplementary Figure 7: An example of calculating the misfit function between data and synthetics for station TA.S14K. The misfit values for each synthetic (red) are calculated within a time window of 20 s before S and 20 s after ScS (shaded region) predicted by IASP91 after waveforms aligned on S peaks. The blue arrows indicate the ScS peaks. Waveforms from top to bottom are synthetics for the LLVP model, LLVP model plus NP ULVZ, and data. The misfit functions are calculated based on the average cross-correlation coefficients (CC), L1-norm (σ_{L1}), and L2-norm (σ_{L2}).

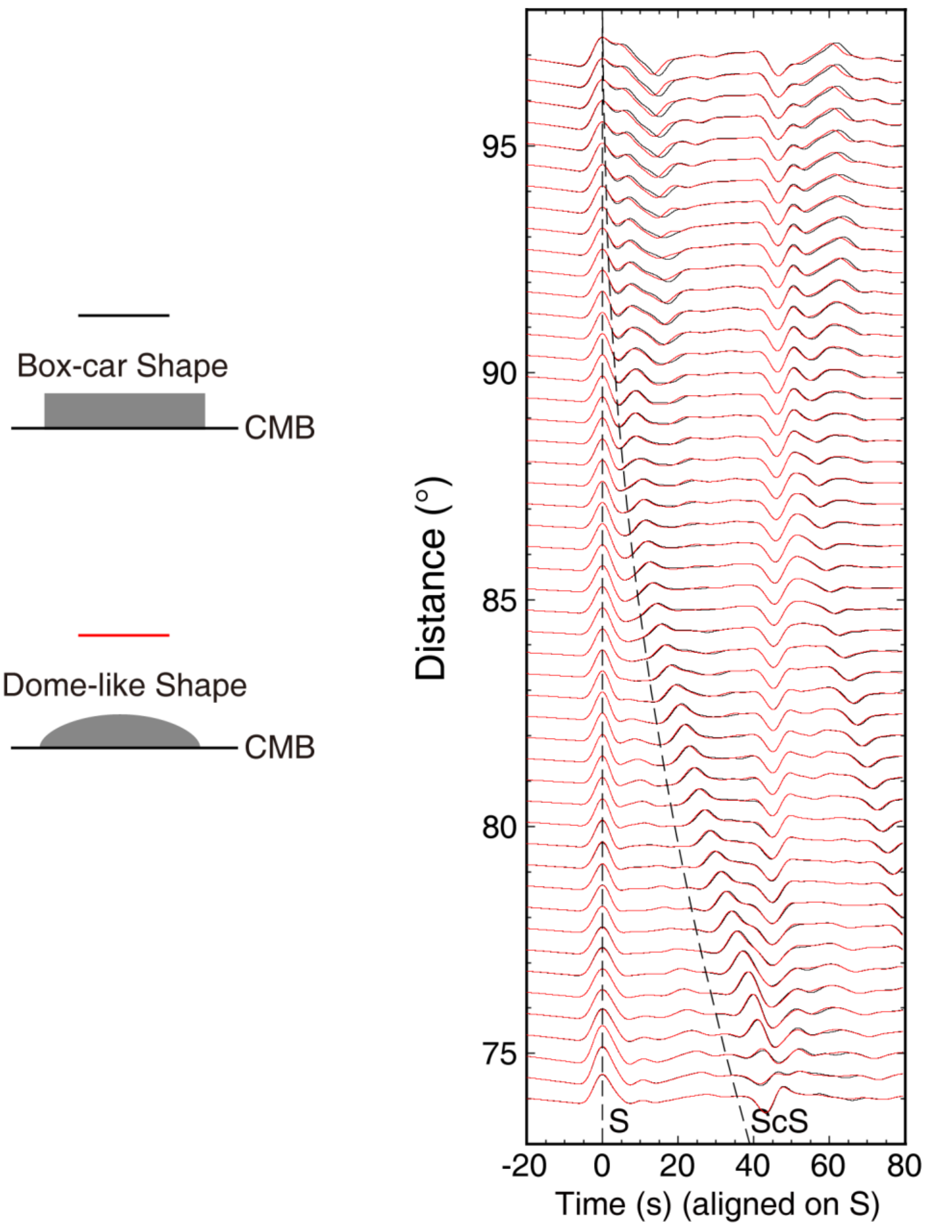

Supplementary Figure 8: Comparison of synthetics from 2D ULVZ models with different shapes. Black lines show synthetic waveforms for a ULVZ with a box-car shape and red lines show waveforms for a ULVZ with a dome-like shape. Focal mechanisms are selected from event B. Dashed lines show the S and ScS arrival times predicted by IASP91[1]. Tangential components are aligned on S and bandpass filtered from 5−50 s. Note the minor difference between the waveforms from the two ULVZ models.

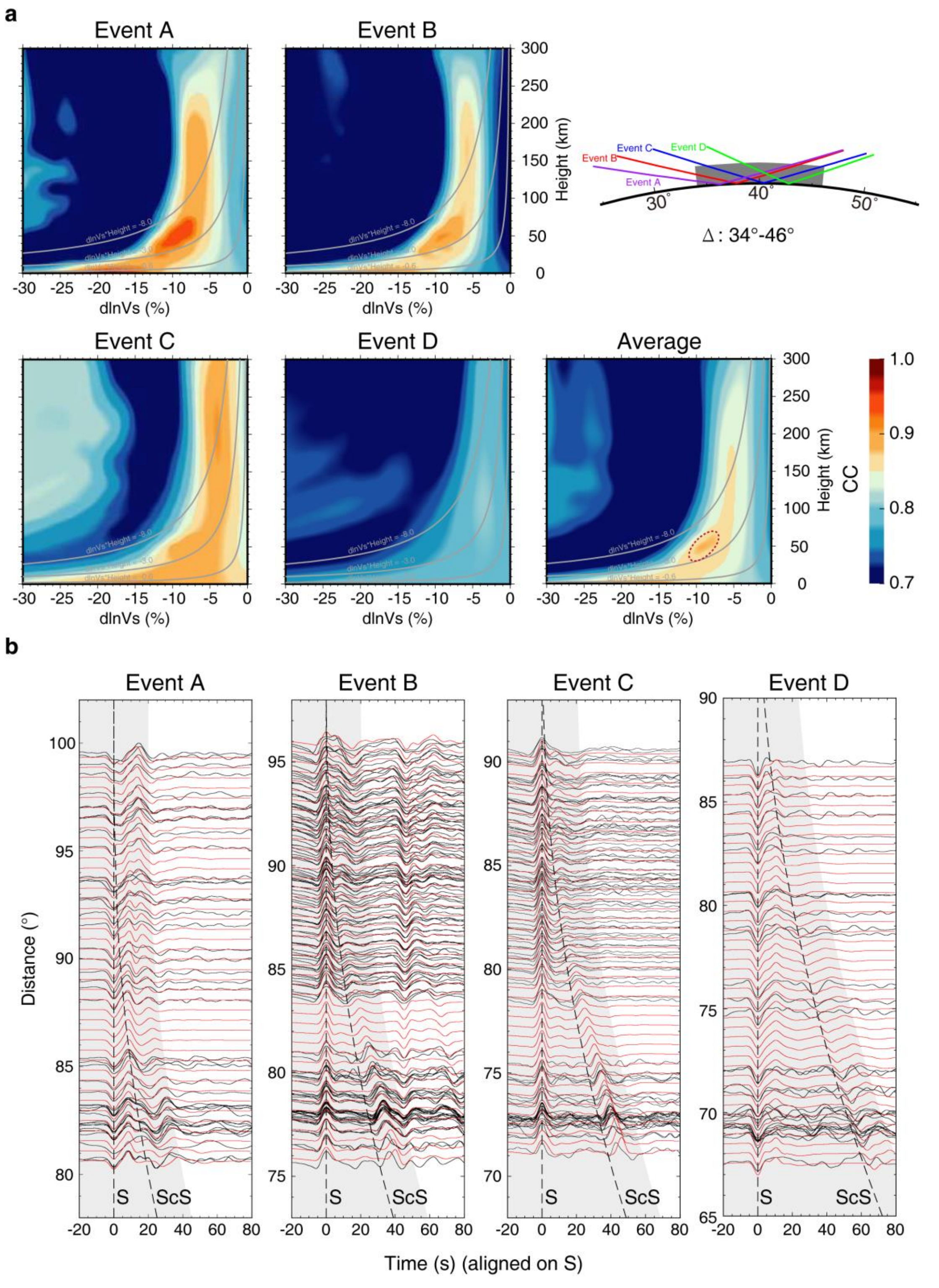

Supplementary Figure 9: Comparison between the data and 2D synthetics of events A−D for distance profiles in 0°−10° azimuth range. a, Trade-off tests between δV_S and H of ULVZ models for events A−D with the ULVZ extending from 34° to 46° (top right panel), using event B as the reference at 0°. Note that the average CC value reaches a maximum (especially for events A and B) when δV_S is about -10% and H is about 50 km (denoted by red dashed circle). **b,** Panels show tangential components

aligned on S for data and synthetics from the ULVZ model with the highest CC in **a** (δV_S = -10%, H = 50 km) which are bandpass filtered from 5–50 s. Predicted arrivals for S and ScS predicted by IASP91[1] are also labeled. Average cross-correlation coefficients (CC) between data and synthetics are calculated in the time window indicated by the grey shaded region. Note similarities between observations and synthetics for waveform complexities, both for move-outs and relative amplitude between S and ScS for all events, as well as sS and sScS for events A and B.

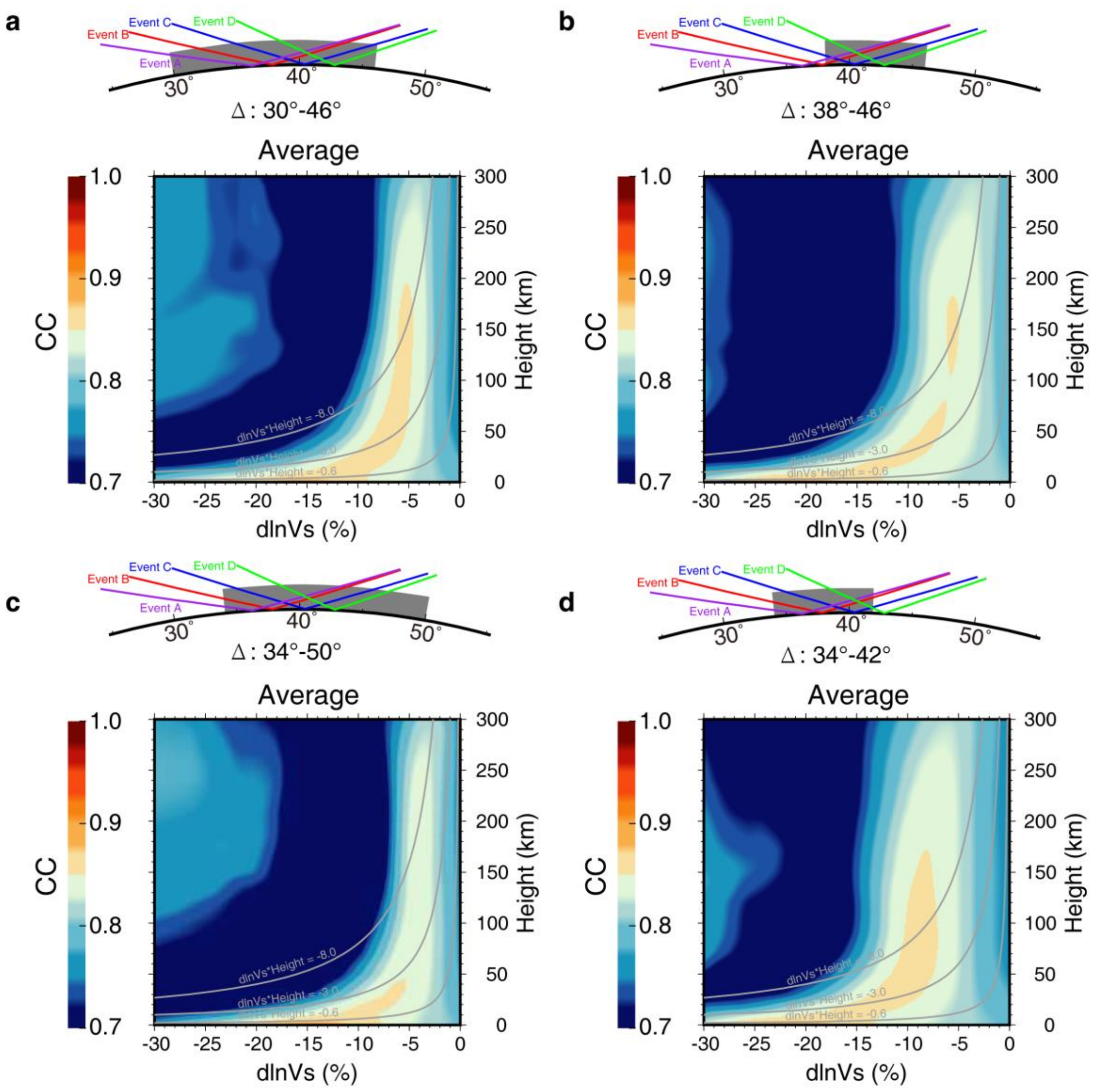

Supplementary Figure 10: Sensitivity tests for the ULVZ model. a, Trade-off tests between δV_S and H of ULVZ models whose extent is presented at the top. Bottom panel shows the average cross-correlation coefficients (CC) of events A–D. **b-d,** Same as **a** but for ULVZ models with different extent.

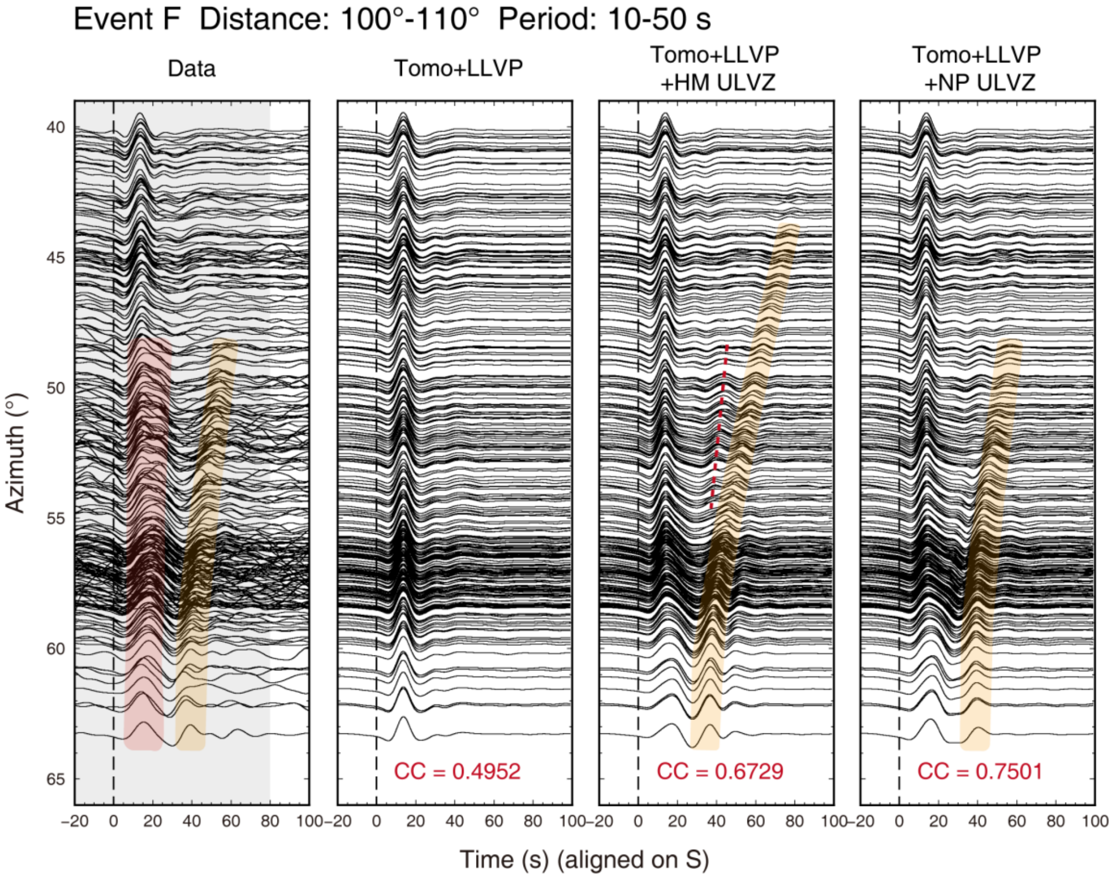

Supplementary Figure 11: Comparison of azimuthal profiles between the data and 3D synthetics of event F in 100°–110° distance range. Tangential components are bandpass filtered from 10–50 s. Columns from left to right are for data, our inferred LLVP model, LLVP model plus HM ULVZ[16], LLVP model plus NP ULVZ, respectively. The yellow patches denote the strong postcursors after Sdiff arrivals. The pink patch denotes two distinct arrivals or broadening of Sdiff arrivals. The red dashed line denotes the double arrivals on the postcursors. For the HM ULVZ model, we observe postcursors extending to the azimuth of 40° and strong extra arrivals leading the postcursors at the azimuth of 48° to 55° (red dash line), which are not presented in the data and synthetics for the NP ULVZ. Average cross-correlation coefficients (CC) and misfits between data and synthetics are calculated in the grey shaded area.

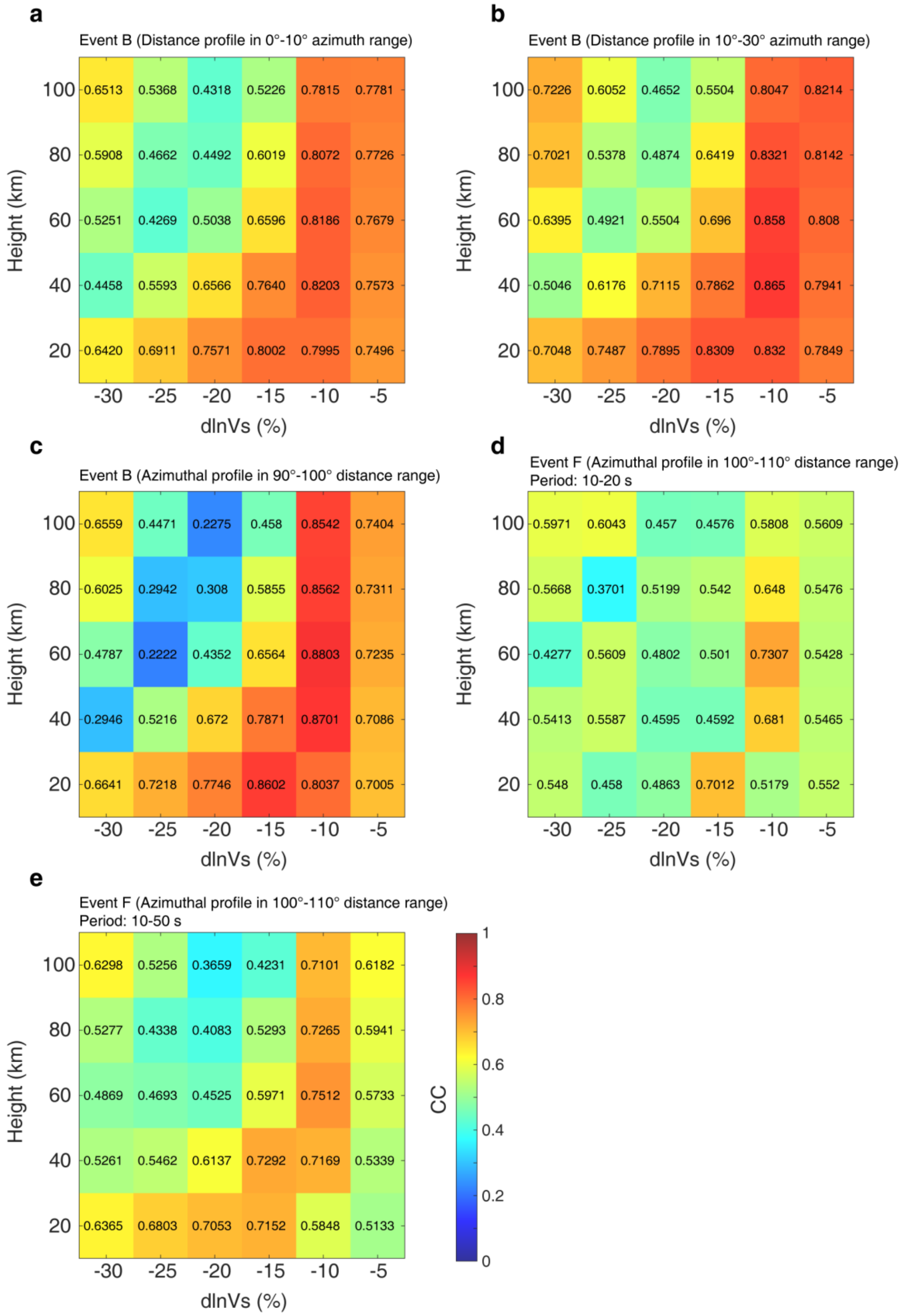

Supplementary Figure 12: Sensitivity tests between δV_S and H for the NP ULVZ model. Average cross-correlation coefficients (CC) are calculated for 5 profiles: **a,** Distance profile in 0°–10° azimuth range of event B (period: 10–50 s); **b,** Distance

profile in 10°–30° azimuth range of event B (period: 10–50 s); **c,** Azimuthal profile in 90°–100° distance range of event B (period: 10–50 s); **d,** Azimuthal profile in 100°–100° distance range of event F (period: 10–20 s); **e,** Azimuthal profile in 100°–100° distance range of event F (period: 10–50 s).

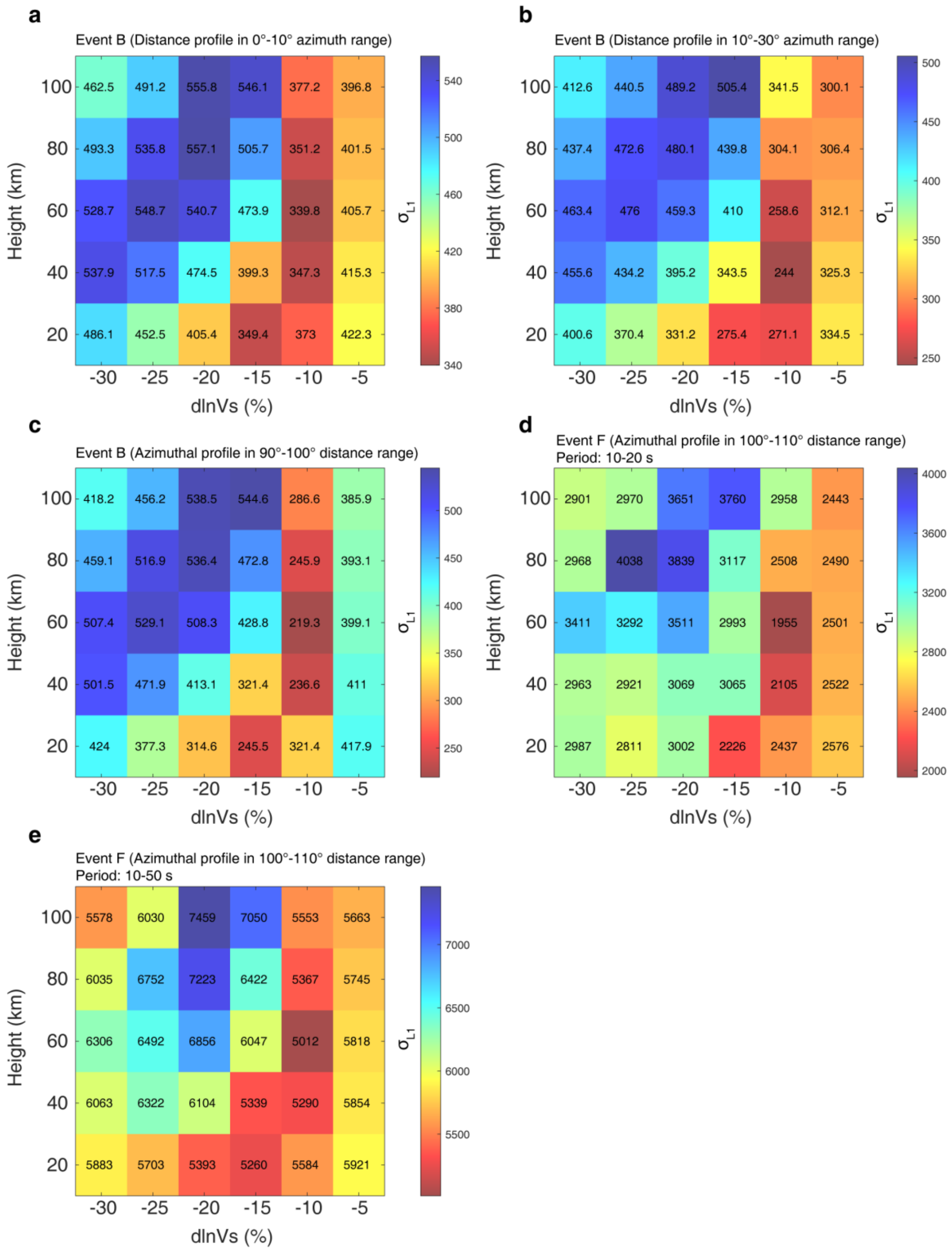

Supplementary Figure 13: **See Supplementary Figure 12 caption, but for L1-norm misfits (σ_{L1}).**

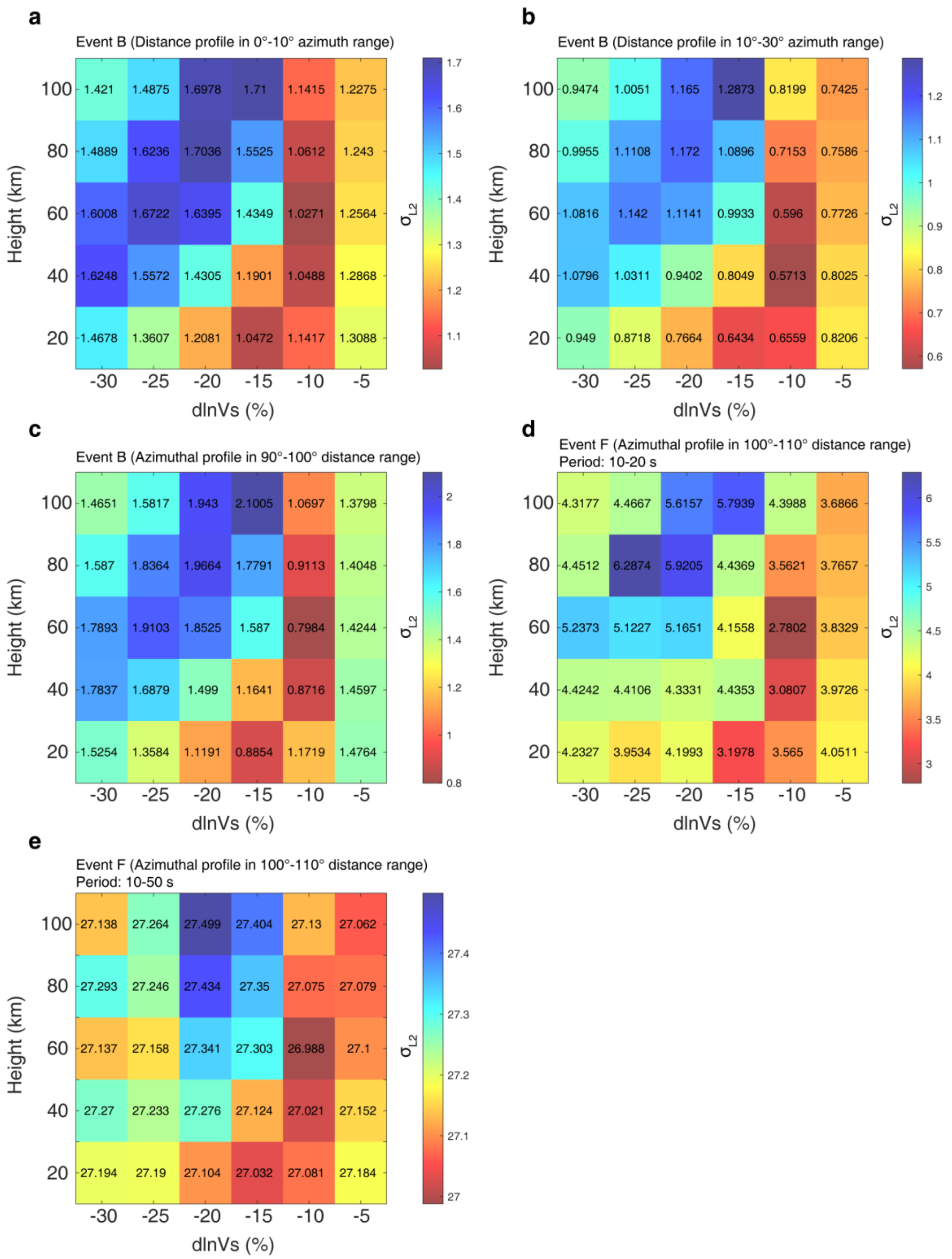

Supplementary Figure 14: **See Supplementary Figure 12 caption, but for L2-norm misfits (σ_{L2}).**

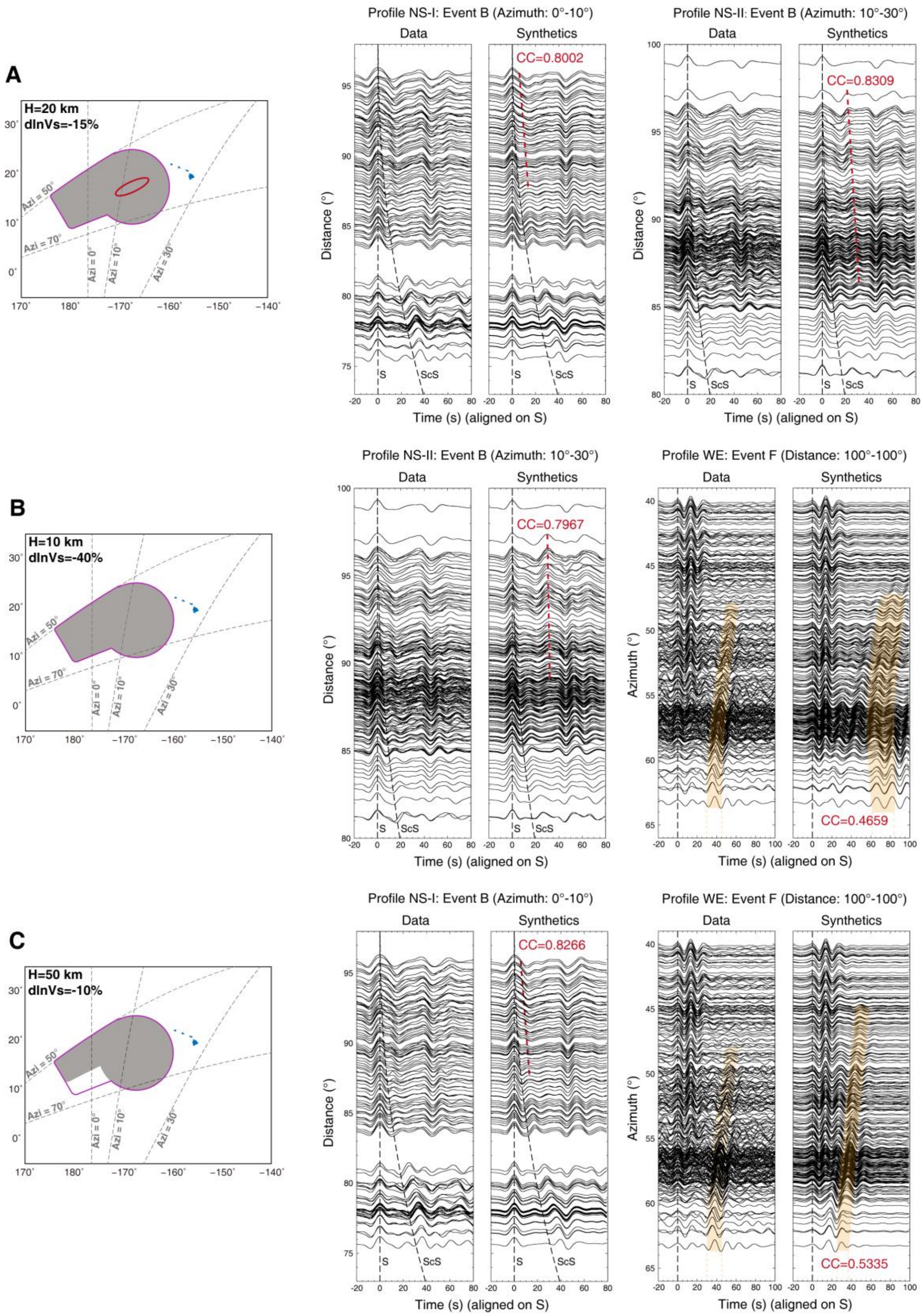
A
H=20 km
dlnVs=-15%
Azi = 50°
Azi = 70°
Azi = 0°
Azi = 10°
Azi = 30°
Profile NS-I: Event B (Azimuth: 0°-10°)
Data
Synthetics
CC=0.8002
Distance (°)
S
ScS
Time (s) (aligned on S)
Profile NS-II: Event B (Azimuth: 10°-30°)
Data
Synthetics
CC=0.8309
Distance (°)
S
ScS
Time (s) (aligned on S)
B
H=10 km
dlnVs=-40%
Profile NS-II: Event B (Azimuth: 10°-30°)
Data
Synthetics
CC=0.7967
Distance (°)
S
ScS
Time (s) (aligned on S)
Profile WE: Event F (Distance: 100°-100°)
Data
Synthetics
Azimuth (°)
CC=0.4659
Time (s) (aligned on S)
C
H=50 km
dlnVs=-10%
Profile NS-I: Event B (Azimuth: 0°-10°)
Data
Synthetics
CC=0.8266
Distance (°)
S
ScS
Time (s) (aligned on S)
Profile WE: Event F (Distance: 100°-100°)
Data
Synthetics
Azimuth (°)
CC=0.5335
Time (s) (aligned on S)

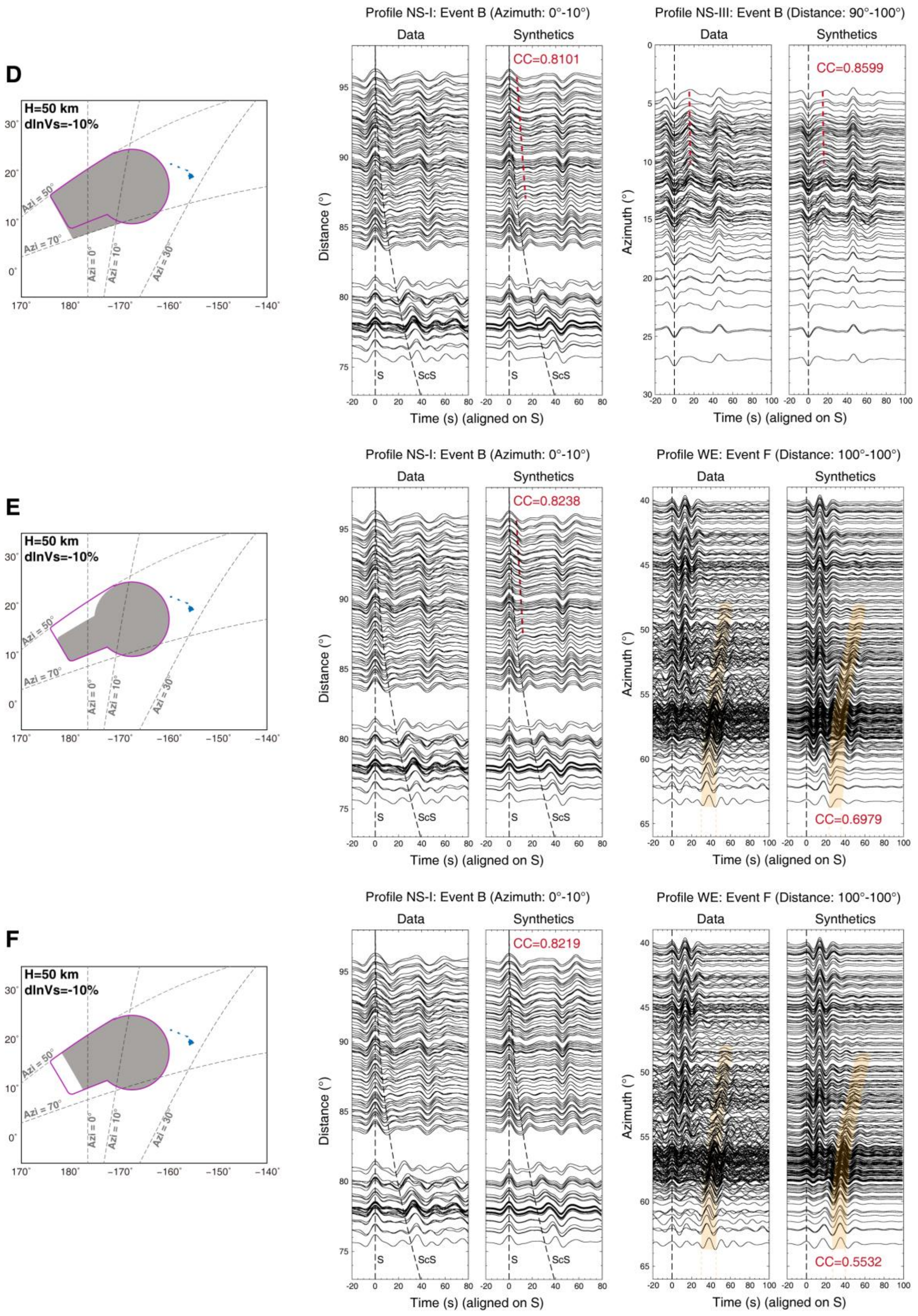
D
H=50 km
dlnVs=-10%
Azi = 50°
Azi = 70°
Azi = 0°
Azi = 10°
Azi = 30°
Profile NS-I: Event B (Azimuth: 0°-10°)
Data
Synthetics
CC=0.8101
Distance (°)
S
ScS
Time (s) (aligned on S)
Profile NS-III: Event B (Distance: 90°-100°)
CC=0.8599
Azimuth (°)
E
H=50 km
dlnVs=-10%
Profile NS-I: Event B (Azimuth: 0°-10°)
CC=0.8238
Profile WE: Event F (Distance: 100°-100°)
CC=0.6979
F
H=50 km
dlnVs=-10%
Profile NS-I: Event B (Azimuth: 0°-10°)
CC=0.8219
Profile WE: Event F (Distance: 100°-100°)
CC=0.5532

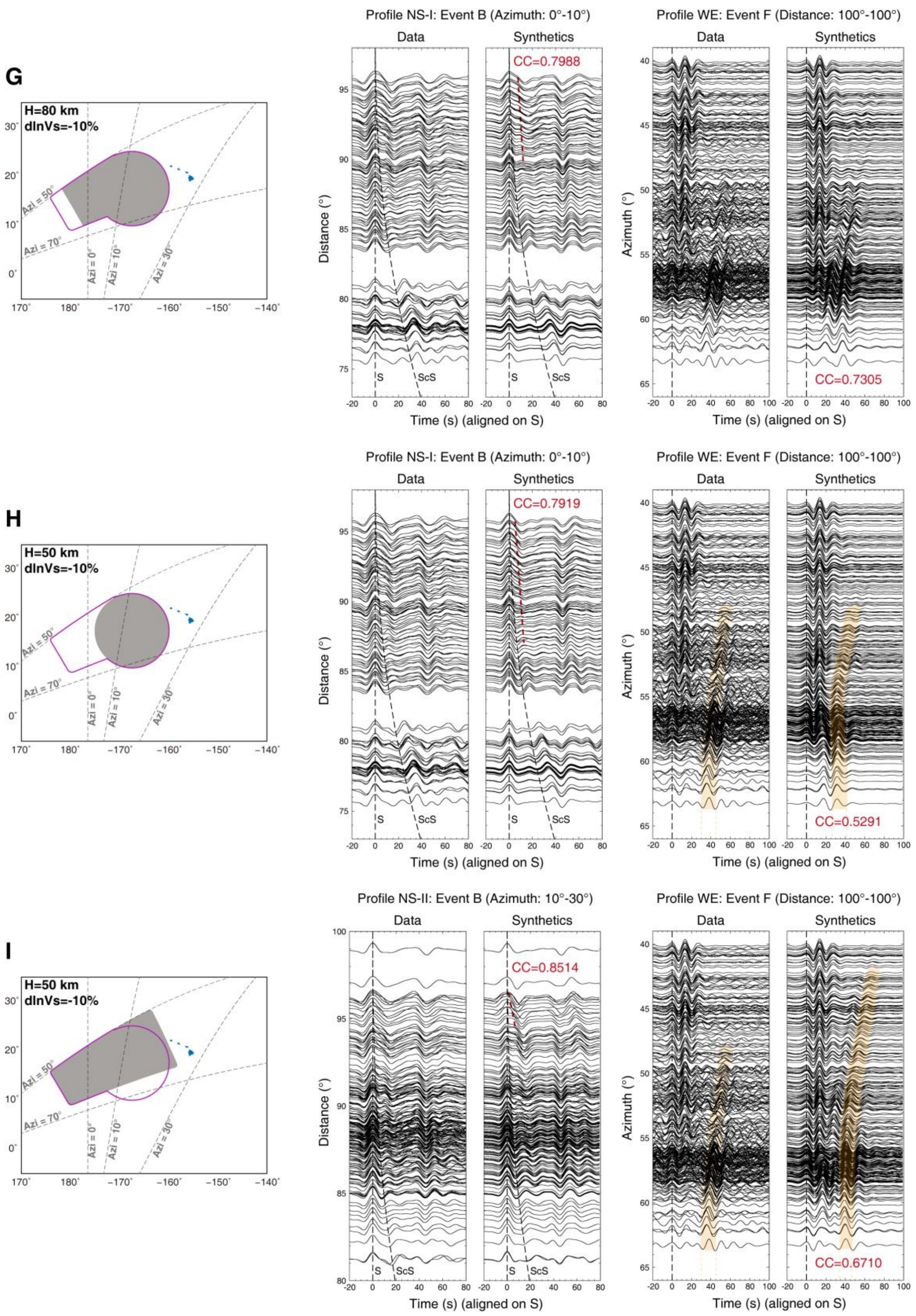
G
H=80 km
dlnVs=-10%
Profile NS-I: Event B (Azimuth: 0°-10°)
Data
Synthetics
CC=0.7988
Profile WE: Event F (Distance: 100°-100°)
Data
Synthetics
CC=0.7305
H
H=50 km
dlnVs=-10%
Profile NS-I: Event B (Azimuth: 0°-10°)
Data
Synthetics
CC=0.7919
Profile WE: Event F (Distance: 100°-100°)
Data
Synthetics
CC=0.5291
I
H=50 km
dlnVs=-10%
Profile NS-II: Event B (Azimuth: 10°-30°)
Data
Synthetics
CC=0.8514
Profile WE: Event F (Distance: 100°-100°)
Data
Synthetics
CC=0.6710
Azi = 50°
Azi = 70°
Azi = 0°
Azi = 10°
Azi = 30°
Distance (°)
Azimuth (°)
S
ScS
Time (s) (aligned on S)

J

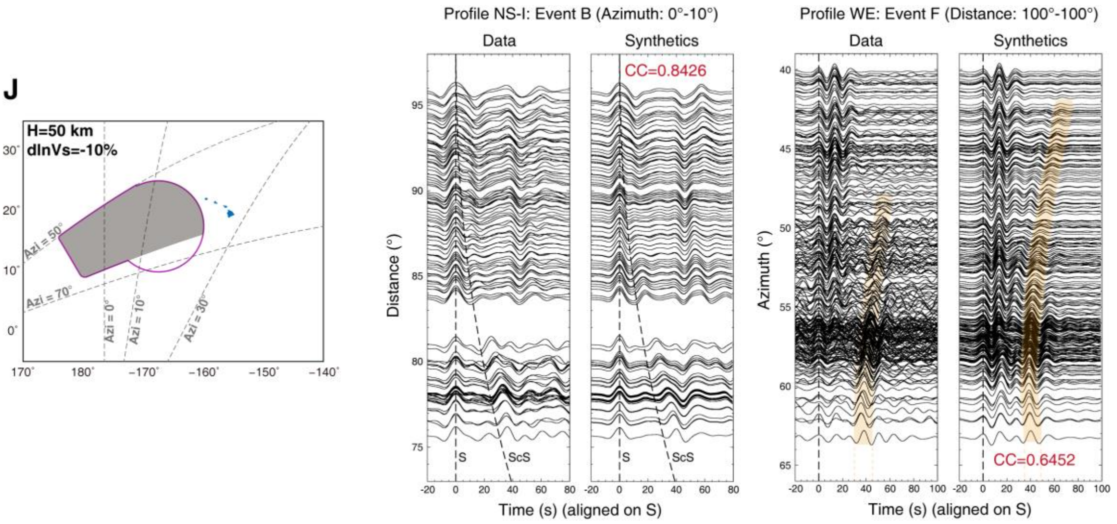
H=50 km
dlnVs=-10%
Azi = 50°
Azi = 70°
Azi = 0°
Azi = 10°
Azi = 30°
Profile NS-I: Event B (Azimuth: 0°-10°)
Data
Synthetics
CC=0.8426
Distance (°)
S
ScS
Time (s) (aligned on S)
Profile WE: Event F (Distance: 100°-100°)
Azimuth (°)
CC=0.6452

K

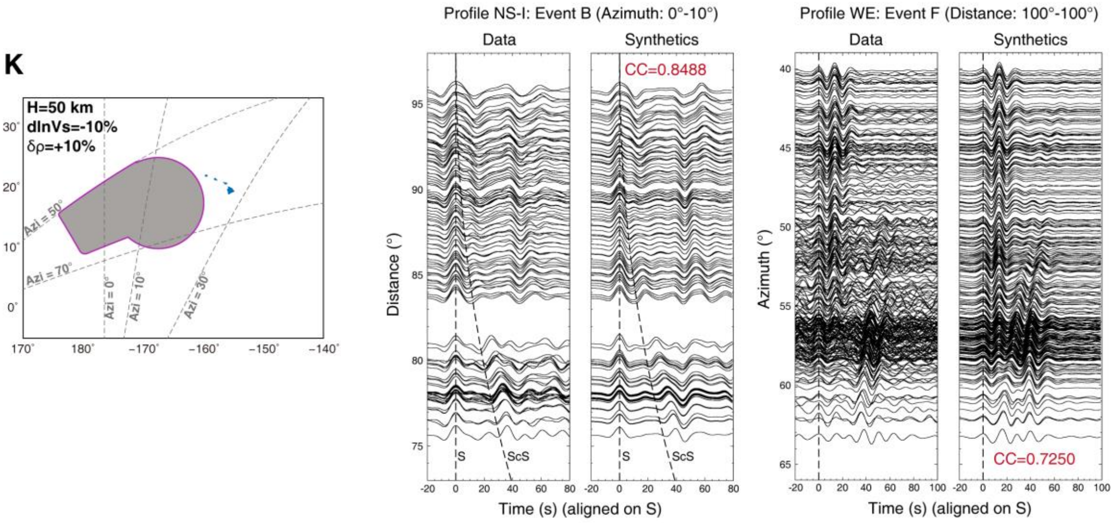
H=50 km
dlnVs=-10%
δρ=+10%
Azi = 50°
Azi = 70°
Azi = 0°
Azi = 10°
Azi = 30°
Profile NS-I: Event B (Azimuth: 0°-10°)
Data
Synthetics
CC=0.8488
Distance (°)
S
ScS
Time (s) (aligned on S)
Profile WE: Event F (Distance: 100°-100°)
Azimuth (°)
CC=0.7250

L

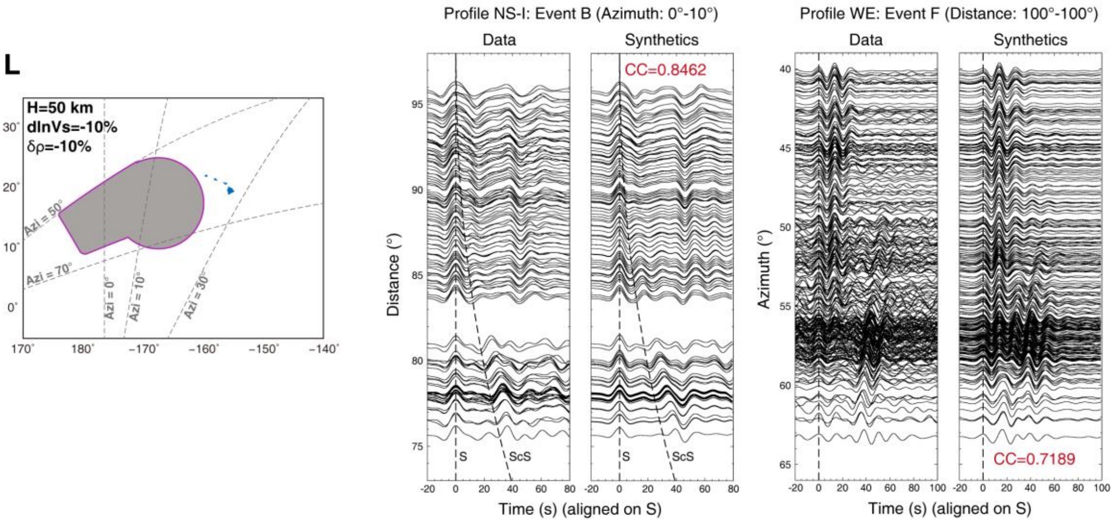
H=50 km
dlnVs=-10%
δρ=-10%
Azi = 50°
Azi = 70°
Azi = 0°
Azi = 10°
Azi = 30°
Profile NS-I: Event B (Azimuth: 0°-10°)
Data
Synthetics
CC=0.8462
Distance (°)
S
ScS
Time (s) (aligned on S)
Profile WE: Event F (Distance: 100°-100°)
Azimuth (°)
CC=0.7189

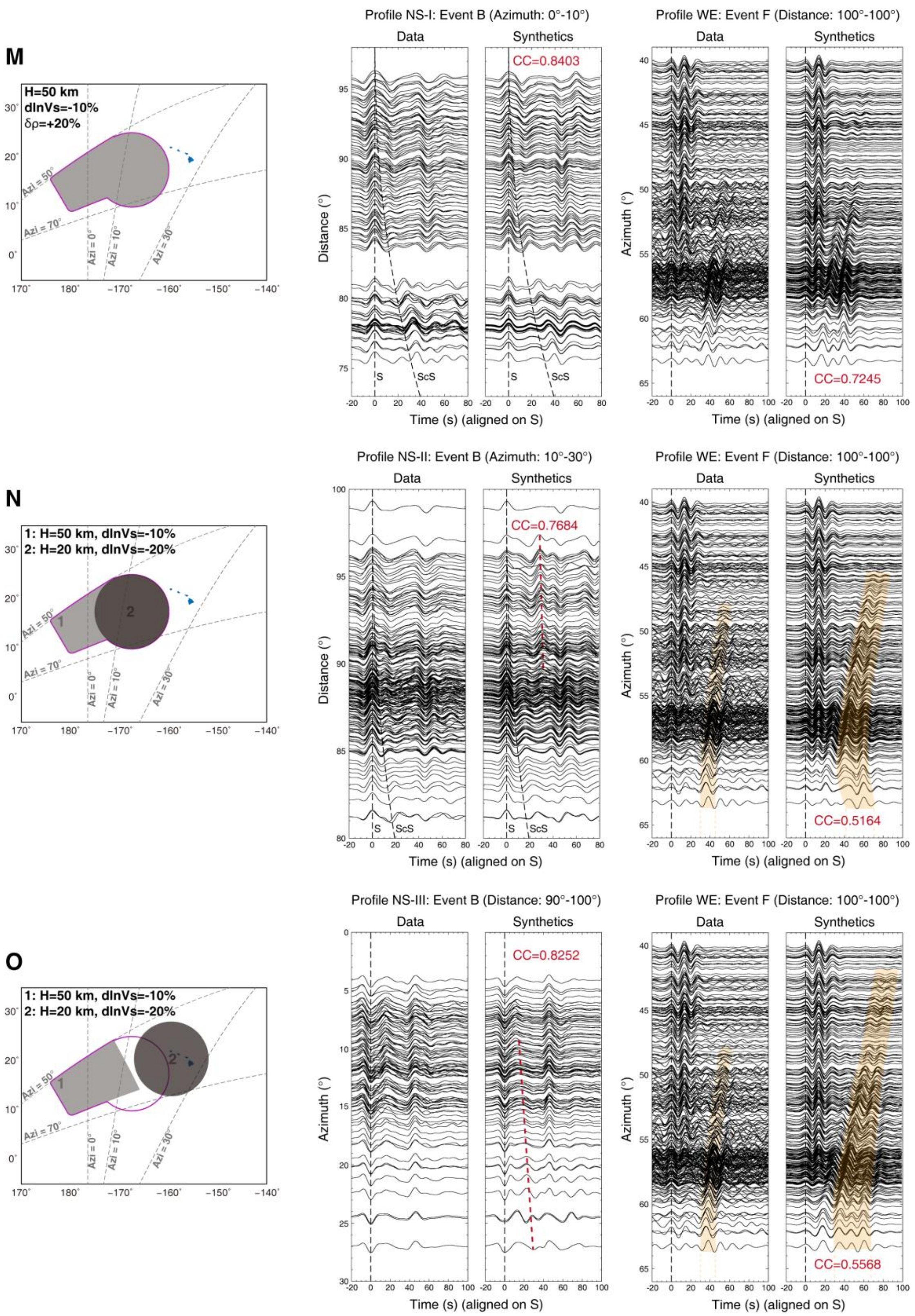
M
H=50 km
dlnVs=-10%
δρ=+20%
Azi = 50°
Azi = 70°
Azi = 0°
Azi = 10°
Azi = 30°
Profile NS-I: Event B (Azimuth: 0°-10°)
Data
Synthetics
CC=0.8403
Distance (°)
S
ScS
Time (s) (aligned on S)
Profile WE: Event F (Distance: 100°-100°)
Azimuth (°)
CC=0.7245
N
1: H=50 km, dlnVs=-10%
2: H=20 km, dlnVs=-20%
Profile NS-II: Event B (Azimuth: 10°-30°)
CC=0.7684
CC=0.5164
O
1: H=50 km, dlnVs=-10%
2: H=20 km, dlnVs=-20%
Profile NS-III: Event B (Distance: 90°-100°)
CC=0.8252
CC=0.5568

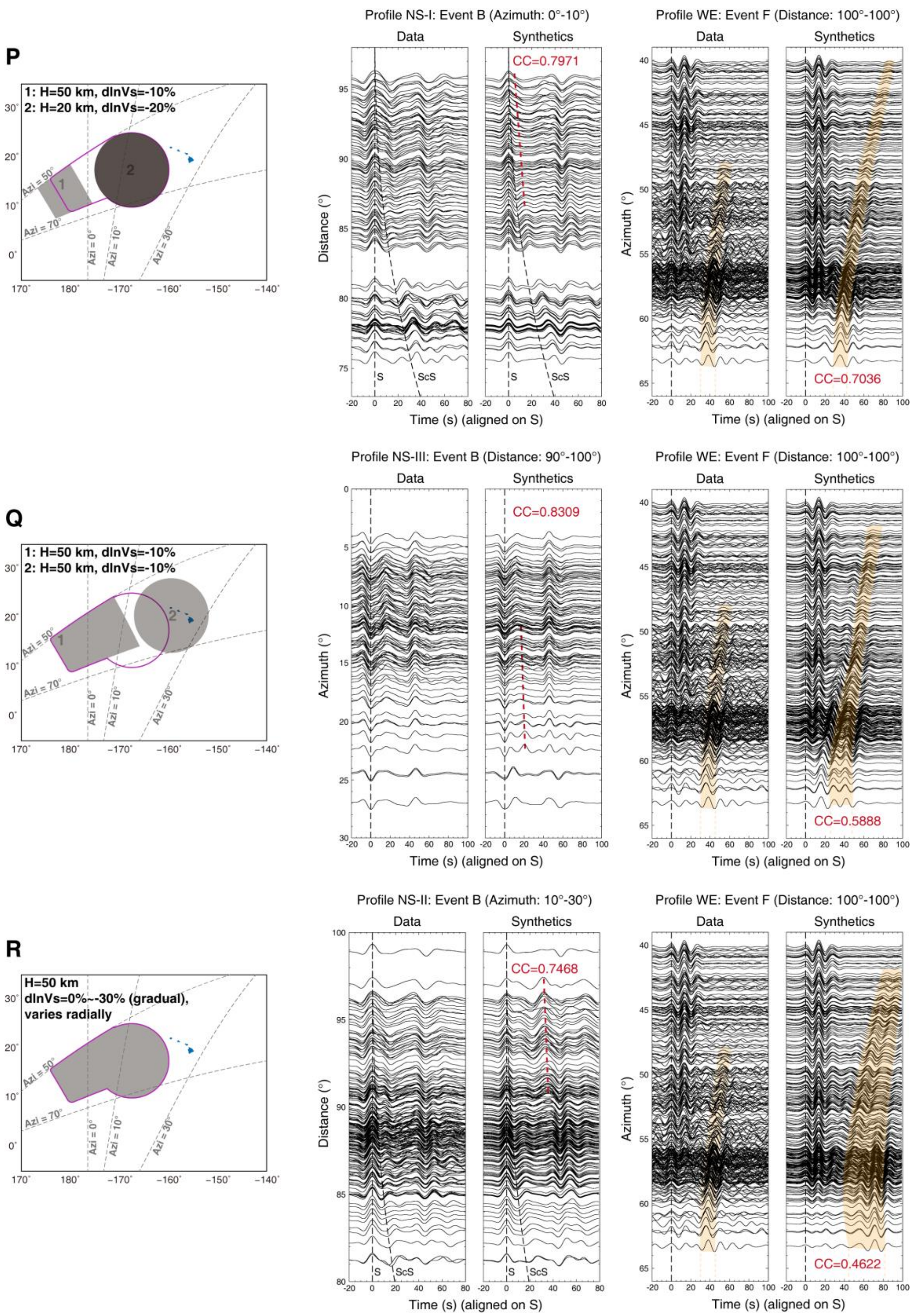
P
1: H=50 km, dlnVs=-10%
2: H=20 km, dlnVs=-20%
Profile NS-I: Event B (Azimuth: 0°-10°)
Data
Synthetics
CC=0.7971
Distance (°)
S
ScS
Time (s) (aligned on S)
Profile WE: Event F (Distance: 100°-100°)
Azimuth (°)
CC=0.7036
Q
1: H=50 km, dlnVs=-10%
2: H=50 km, dlnVs=-10%
Profile NS-III: Event B (Distance: 90°-100°)
CC=0.8309
CC=0.5888
R
H=50 km
dlnVs=0%~-30% (gradual),
varies radially
Profile NS-II: Event B (Azimuth: 10°-30°)
CC=0.7468
CC=0.4622
Azi = 0°
Azi = 10°
Azi = 30°
Azi = 50°
Azi = 70°

S

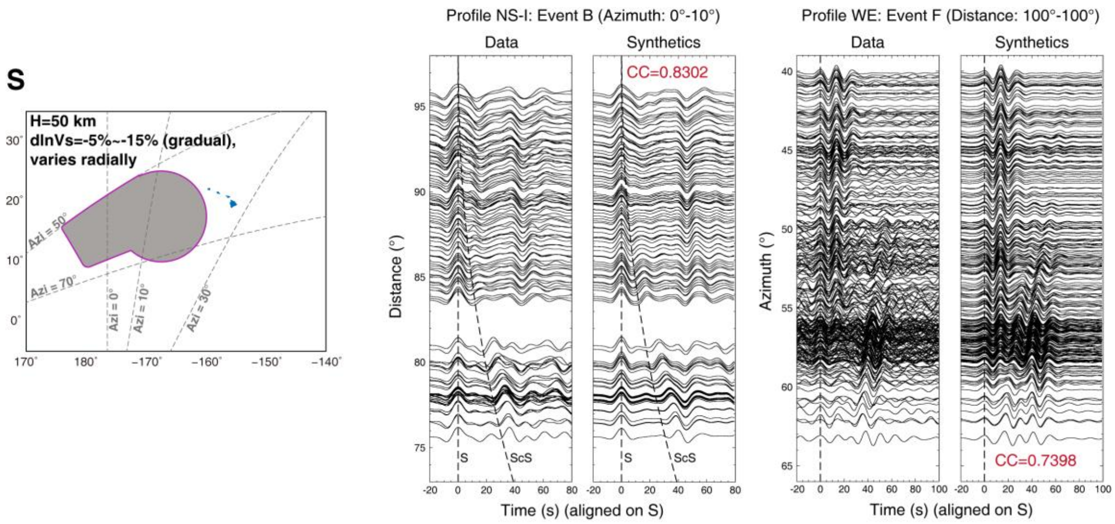
H=50 km
dlnVs=-5%~-15% (gradual), varies radially
Azi = 50°
Azi = 70°
Azi = 0°
Azi = 10°
Azi = 30°
Profile NS-I: Event B (Azimuth: 0°-10°)
Data
Synthetics
CC=0.8302
Distance (°)
S
ScS
Time (s) (aligned on S)
Profile WE: Event F (Distance: 100°-100°)
Azimuth (°)
CC=0.7398

T

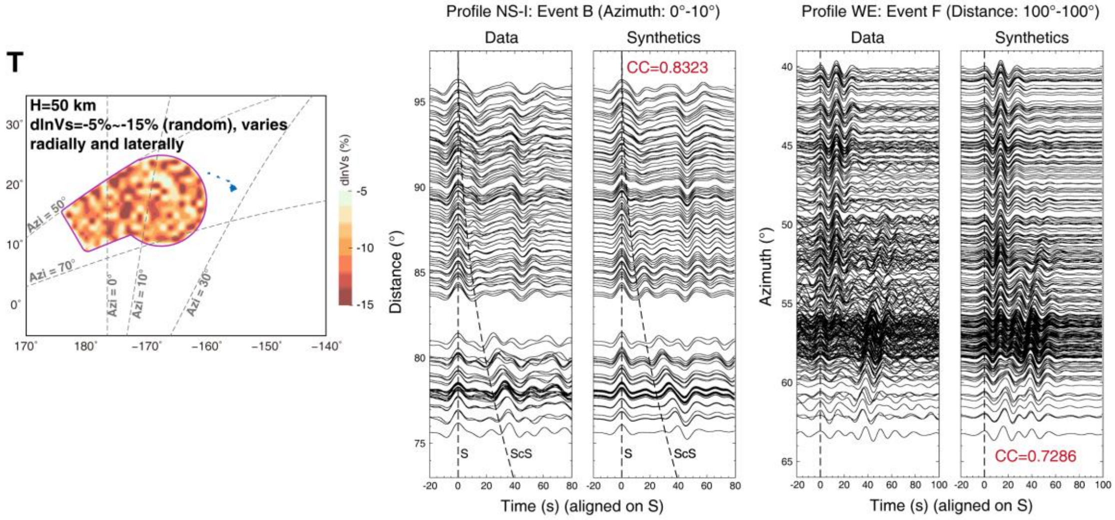
H=50 km
dlnVs=-5%~-15% (random), varies radially and laterally
dlnVs (%)
Azi = 50°
Azi = 70°
Azi = 0°
Azi = 10°
Azi = 30°
Profile NS-I: Event B (Azimuth: 0°-10°)
Data
Synthetics
CC=0.8323
Distance (°)
S
ScS
Time (s) (aligned on S)
Profile WE: Event F (Distance: 100°-100°)
Azimuth (°)
CC=0.7286

U

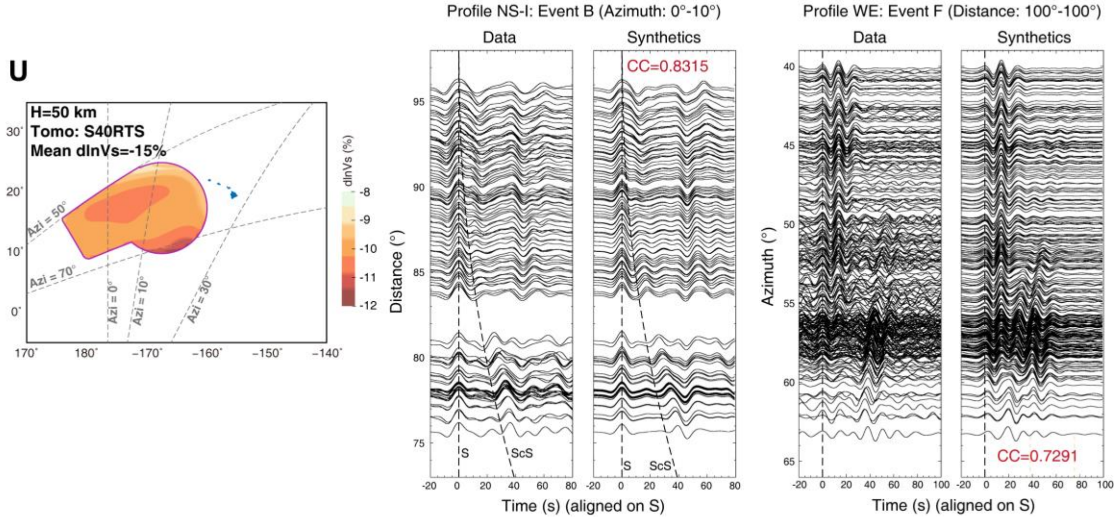
H=50 km
Tomo: S40RTS
Mean dlnVs=-15%
dlnVs (%)
Azi = 50°
Azi = 70°
Azi = 0°
Azi = 10°
Azi = 30°
Profile NS-I: Event B (Azimuth: 0°-10°)
Data
Synthetics
CC=0.8315
Distance (°)
S
ScS
Time (s) (aligned on S)
Profile WE: Event F (Distance: 100°-100°)
Azimuth (°)
CC=0.7291

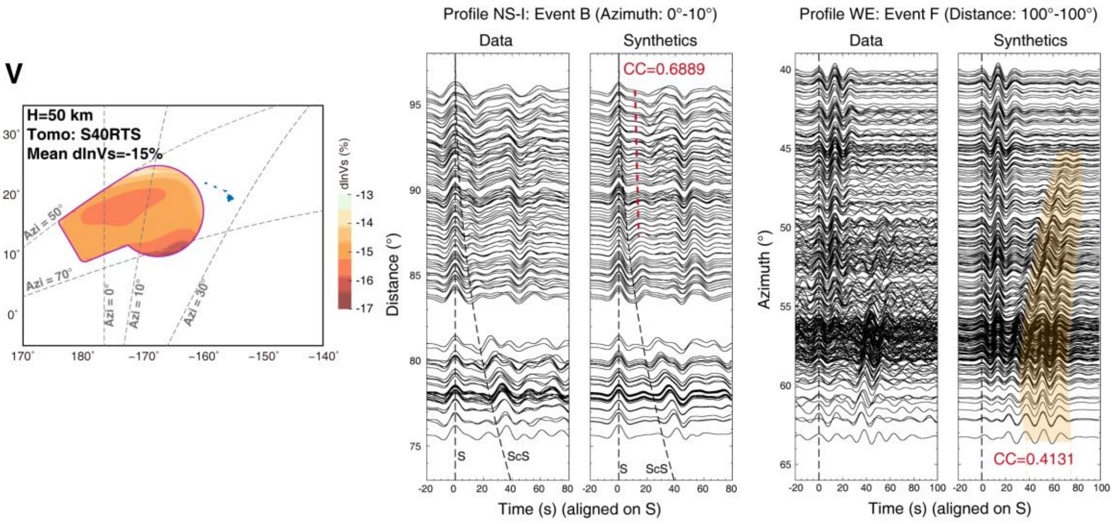
V
H=50 km
Tomo: S40RTS
Mean dlnVs=-15%
dlnVs (%)
Azi = 50°
Azi = 70°
Azi = 0°
Azi = 10°
Azi = 30°
Profile NS-I: Event B (Azimuth: 0°-10°)
Data
Synthetics
CC=0.6889
Distance (°)
S
ScS
Time (s) (aligned on S)
Profile WE: Event F (Distance: 100°-100°)
Azimuth (°)
CC=0.4131

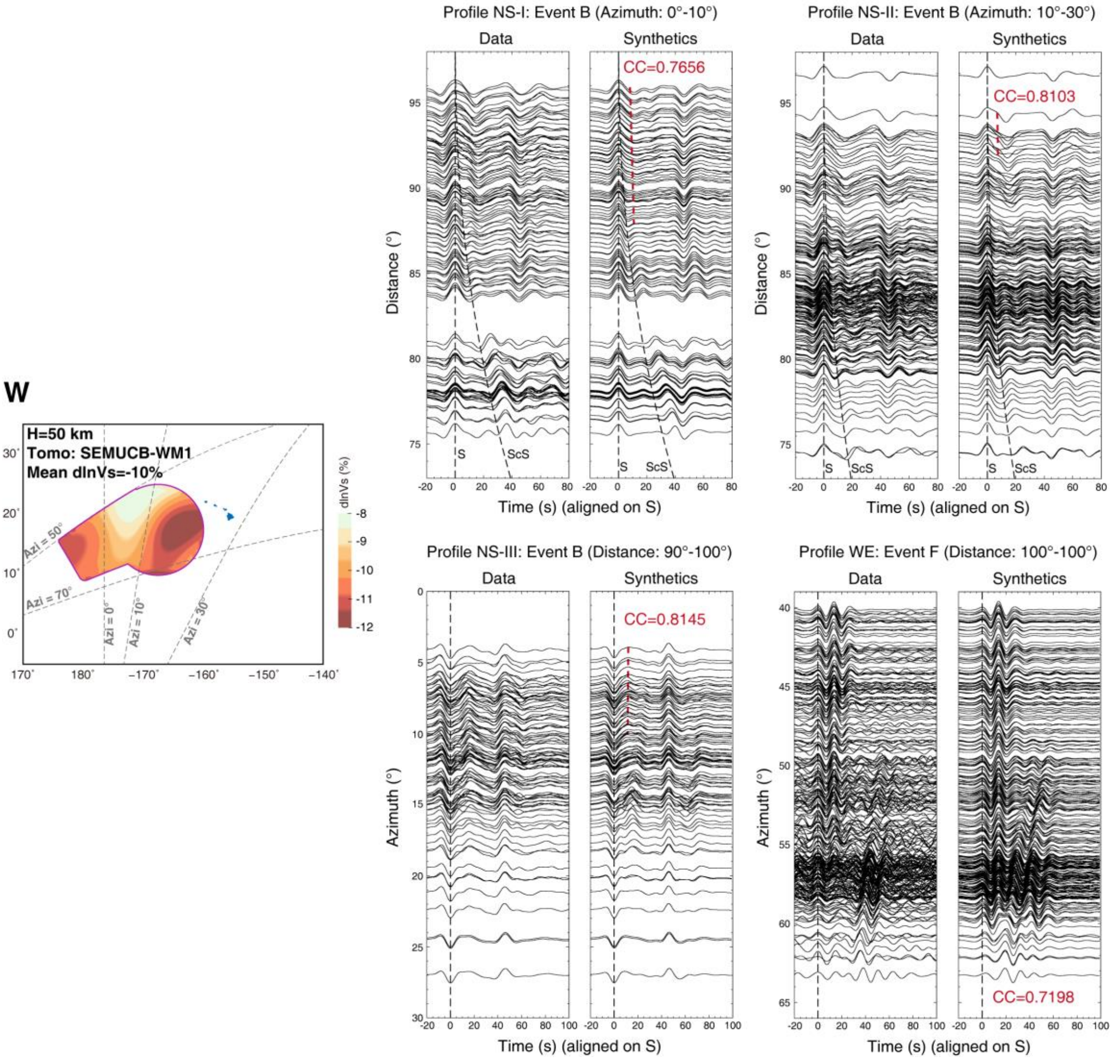
W
H=50 km
Tomo: SEMUCB-WM1
Mean dlnVs=-10%
dlnVs (%)
Azi = 50°
Azi = 70°
Azi = 0°
Azi = 10°
Azi = 30°
Profile NS-I: Event B (Azimuth: 0°-10°)
Data
Synthetics
CC=0.7656
Distance (°)
S
ScS
Time (s) (aligned on S)
Profile NS-II: Event B (Azimuth: 10°-30°)
CC=0.8103
Profile NS-III: Event B (Distance: 90°-100°)
Azimuth (°)
CC=0.8145
Profile WE: Event F (Distance: 100°-100°)
CC=0.7198

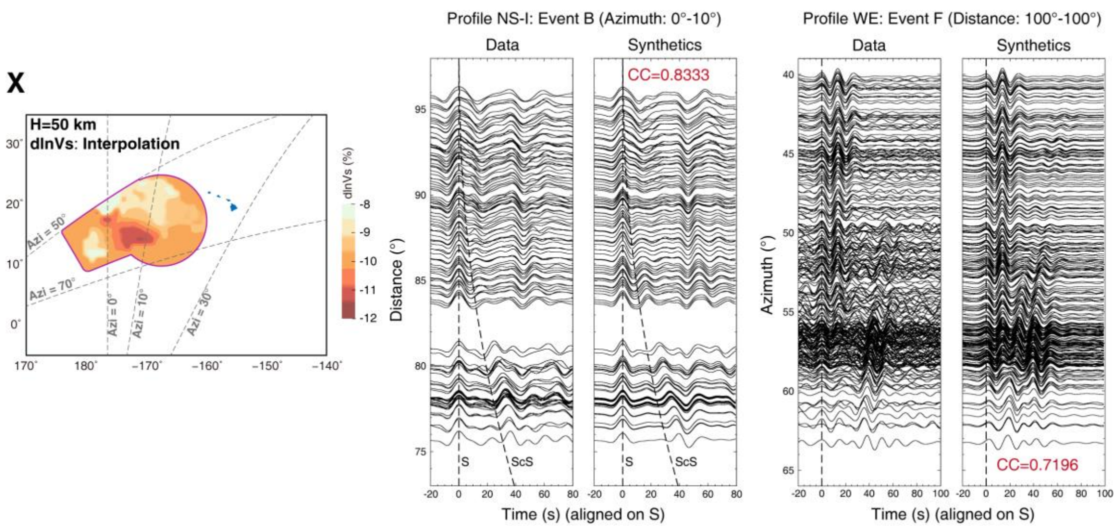
X
H=50 km
dlnVs: Interpolation
dlnVs (%)
Azi = 50°
Azi = 70°
Azi = 0°
Azi = 10°
Azi = 30°
Profile NS-I: Event B (Azimuth: 0°-10°)
Data
Synthetics
CC=0.8333
Distance (°)
S
ScS
Time (s) (aligned on S)
Profile WE: Event F (Distance: 100°-100°)
Azimuth (°)
CC=0.7196

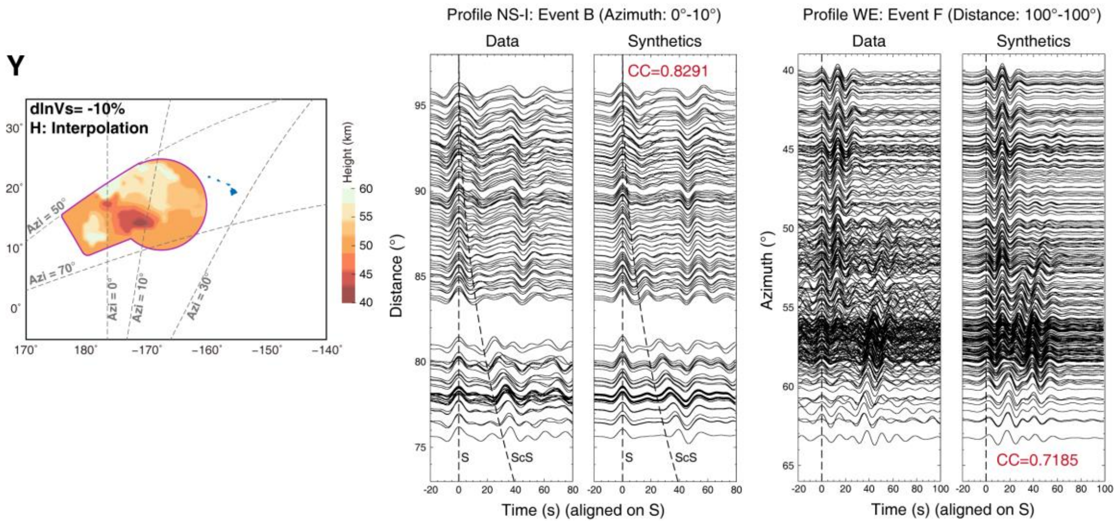
Y
dlnVs= -10%
H: Interpolation
Height (km)
Azi = 50°
Azi = 70°
Azi = 0°
Azi = 10°
Azi = 30°
Profile NS-I: Event B (Azimuth: 0°-10°)
Data
Synthetics
CC=0.8291
Distance (°)
S
ScS
Time (s) (aligned on S)
Profile WE: Event F (Distance: 100°-100°)
Azimuth (°)
CC=0.7185

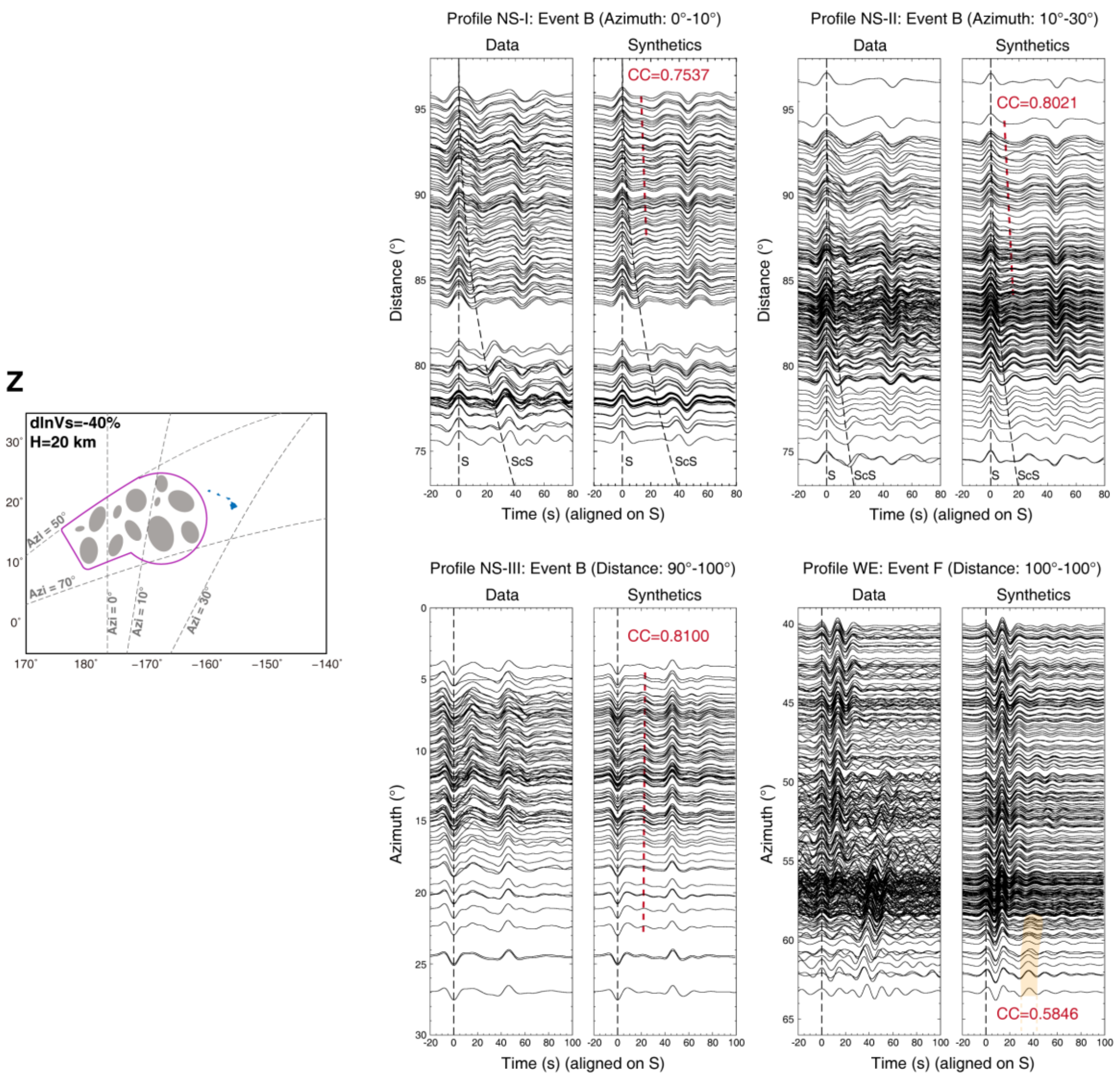

Supplementary Figure 15: Sensitivity tests for the NP ULVZ model. The left panels show the geometries of the ULVZ models. The purple outline represents our best NP ULVZ model while the shaded area represents an alternative ULVZ model for which synthetics are computed in the right panels. The gray dashed lines mark different azimuths for events B and F. The right panels only show profiles in which waveforms are most affected by the ULVZ model indicated by red dashed lines or yellow patches selected from profiles NS-I, NS-II, NS-III and profile WE. Profile NS-I: Comparison of distance profiles between the data and 3D synthetics of event B in 0°–10° azimuth range. Tangential components are aligned on S and are bandpass filtered from 10–50 s. The dashed lines show the S and ScS arrival times predicted by IASP91[1]. Profile NS-II: Same as Profile I but for data in 10°–30° azimuth range. Profile NS-III: Comparison of azimuthal profiles between the data and 3D synthetics of event B in 90°–100° distance range, in which the differential travel time between S and ScS are mainly caused by the ULVZ as ray paths of S and ScS are extremely close to each other. Tangential components are aligned on S and are bandpass filtered from 10–50 s. Profile WE: Comparison of azimuthal profiles between the data and 3D synthetics of event F in 100°–110° distance range. Tangential components are bandpass filtered from 10–20

s. The average cross-correlation coefficients (CC) are given. Sensitivity tests are computed for: A-B) δV_S and H; C-D) southern boundary of the western side; E) northern boundary of the western side; F-G) western boundary; H-J) shape; K-M) density; N-Z) non-uniform δV_S. The red ellipse in A represents the Fresnel Zone of Sdiff for 10 s period along 60° azimuth of event F.

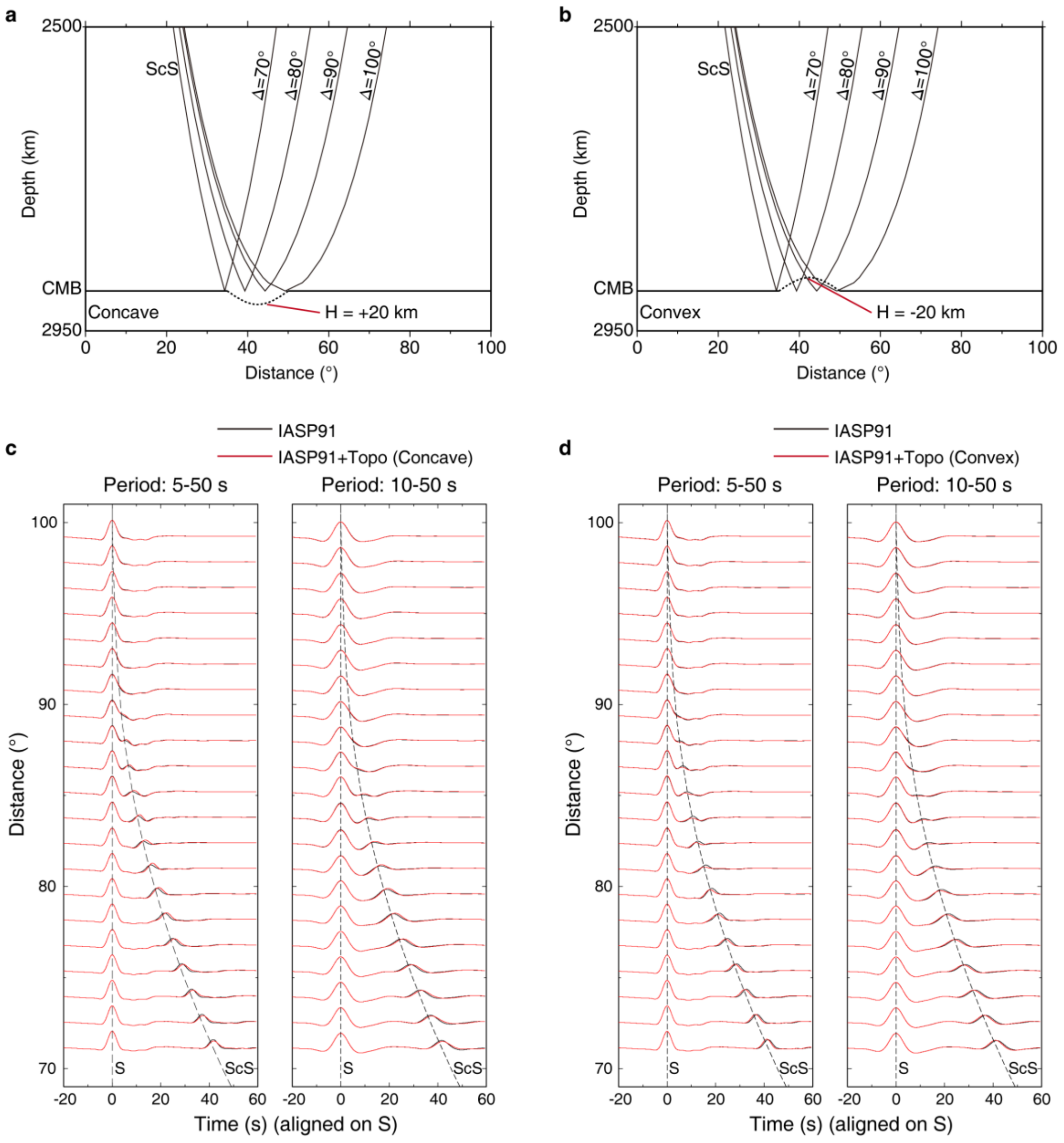

Supplementary Figure 16: Effects of 2D CMB topography on waveforms. a-b, Models with CMB topography and the related ScS ray paths. The shape of the topography variation is assumed to be axial symmetric. The width of the region having topography (L) is 15°, which is comparable to the size of the NP ULVZ. The relief topography (H) is 20 km. **c-d,** Panels show synthetics with (red) and without (black) CMB topography. All synthetics are aligned on S and bandpass filtered from 5−50 s (left) and 10−50 s (right). The dashed lines show the S and ScS arrival times predicted by IASP91[1].

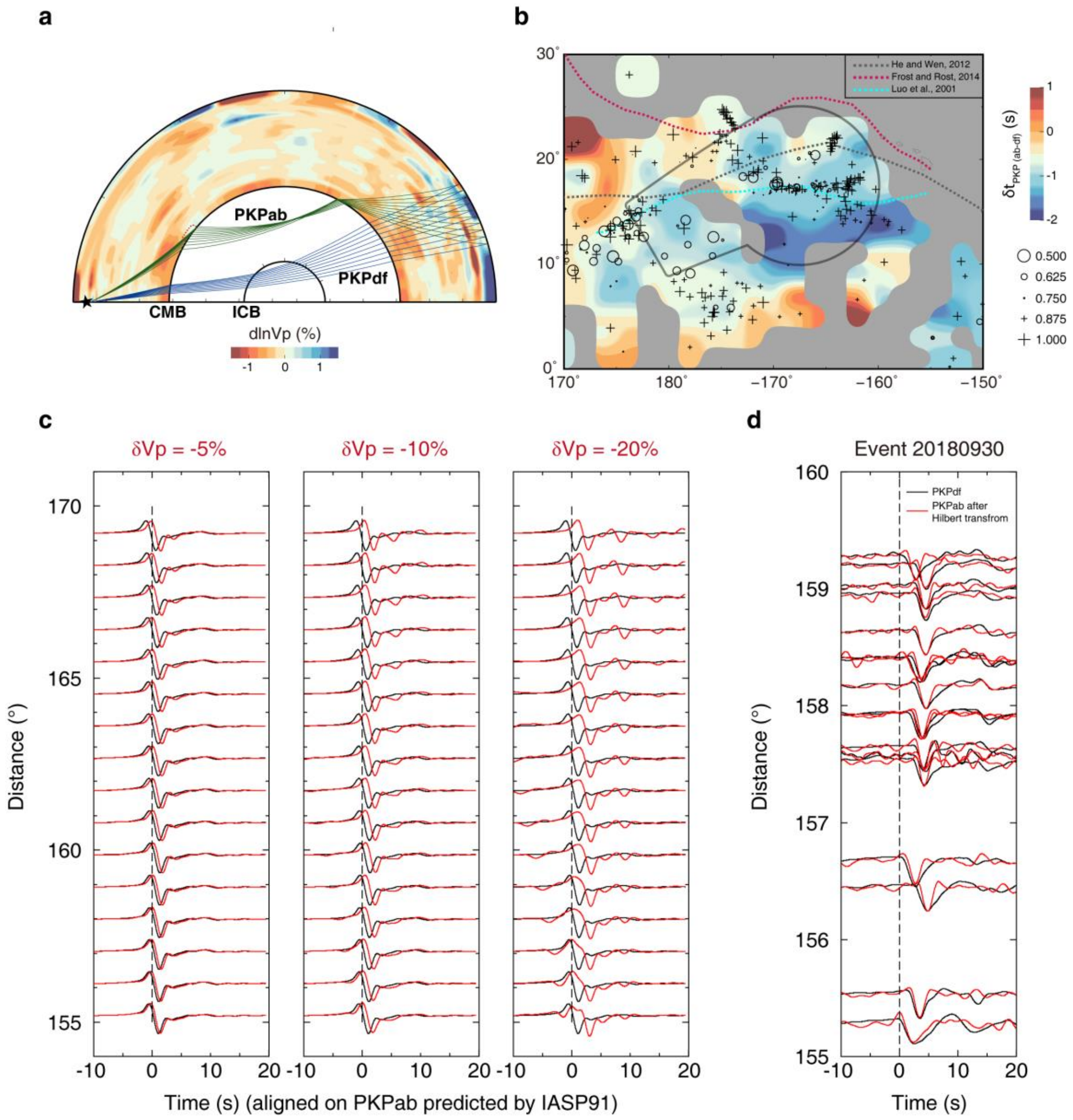

Supplementary Figure 17: Differential travel time residuals ($\delta t_{PKP(ab\text{-}df)}$) relative to IASP91[1] and synthetics. a, Ray paths of PKPab (green) and PKPdf (blue) against the GyPSuM P-wave tomography model[6]. The depth cross-section is chosen from Fiji to Europe. **b,** Interpolated $\delta t_{PKP(ab\text{-}df)}$ results with cross-correlation coefficients denoted by circles and crosses. The cross-correlation coefficients are normalized to the maximum for each event. The black outline represents the NP ULVZ model. **c,** Synthetics for the NP ULVZ model with red for $\boldsymbol{\delta V_P}$ = -5%, -10% and -20% and black for GyPSuM aligned on PKPab predicted by IASP91. **d,** Displacement seismograms of event 20180930 with black for PKPdf and red for PKPab after Hilbert transform imposed on PKPdf to show high similarity between them. Data and synthetics are bandpass filtered from 1–50 s.

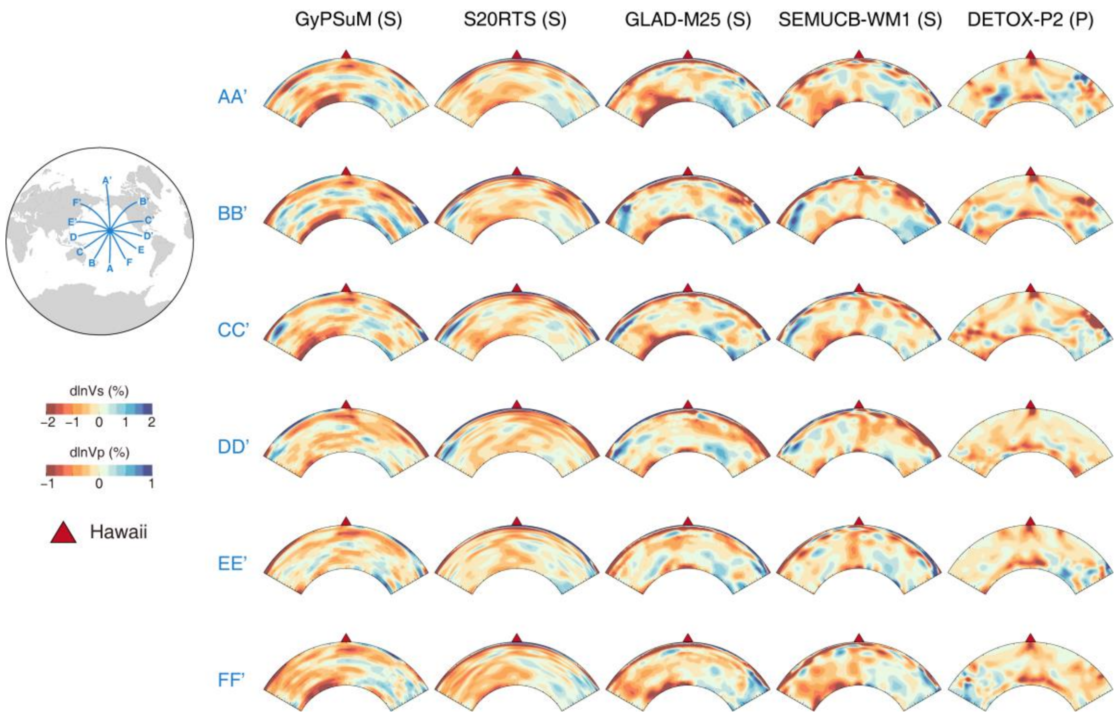

Supplementary Figure 18: Cross-sections through Hawaii for several tomography models. Columns from left to right are for GyPSuM[6] (S), S20RTS[9] (S), GLAD-M25[10] (S), SEMUCB-WM1[3] (S), DETOX-P2[11] (P). Six profiles (AA', BB', CC', DD', EE', FF') are plotted.

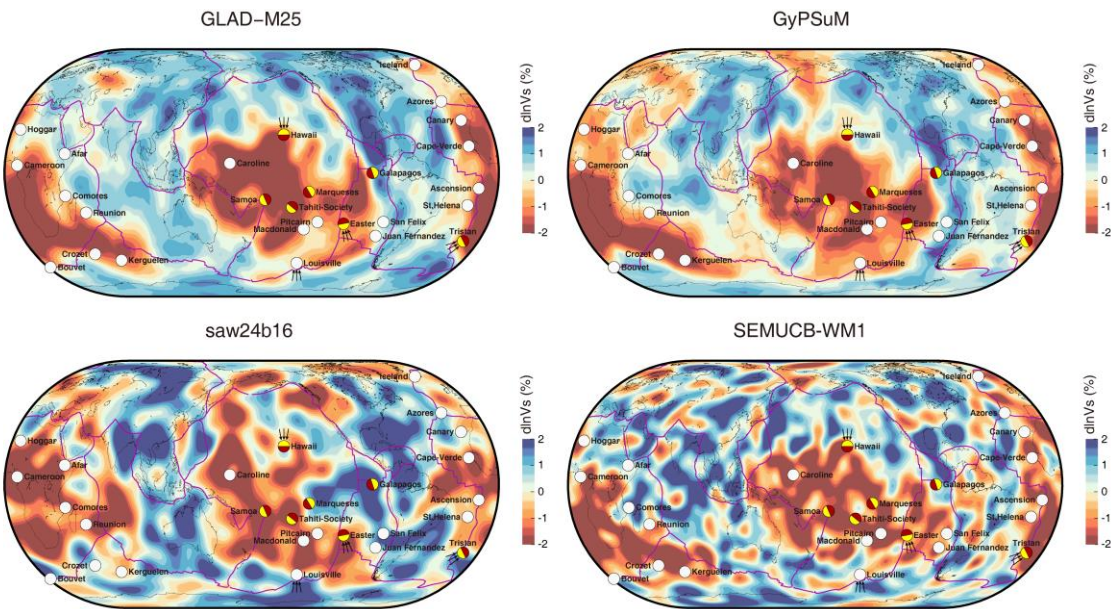

Supplementary Figure 19: Relationship between the deep-rooted plumes and LLVP boundaries. Tomographic images are for GLAD-M25[10], GyPSuM[6], saw24b16[17] and SEMUCB-WM1[3] at the CMB. Deep-rooted plumes identified by French and Romanowicz[3] are plotted as white circles. Some deep-rooted plumes display bilateral zonation with enriched (red) and depleted (yellow) composition oriented towards and away from the LLVP interiors[14,15]. Mantle flow vectors identified by Hassan et al.[18] are superimposed on four plumes (Hawaii, Easter, Louisville, and Tristan).

Event ID	Profile	Date	Lat (°)	Lon (°)	Depth (km)
A[T]	NS	2016/07/13	-28.05	-176.27	12
B[T]	NS	2017/09/26	-23.79	-176.47	110
C[T]	NS	2018/09/30	-18.33	-178.06	566
D[T]	NS	2016/07/10	-15.06	-172.95	8
E[T]	NS	2018/09/10	-31.91	-179.13	119
F[S]	WE	2010/03/20	-3.32	152.33	413
G[T]	NS	2017/06/29	-30.93	-179.92	404
H[T]	NS	2018/04/02	-24.73	-176.73	92
I[T]	NS	2017/06/17	-24.15	179.66	10
J[T]	NS	2017/02/24	-23.44	-178.77	417
K[T]	NS	2016/05/28	-22.13	-179.17	405
L[T]	NS	2016/05/27	-20.77	-178.61	567
M[T]	NS	2016/09/24	-19.77	-178.15	596
N[T]	NS	2017/11/04	-15.04	-173.06	34
O[S]	WE	2016/08/31	-3.58	152.86	490
P[S]	WE	2013/07/07	-4.10	153.98	382
Q[S]	WE	2003/06/12	-5.97	154.95	185
R[S]	WE	2017/01/22	-6.03	154.94	149
S[S]	WE	2019/01/26	-6.99	156.38	364

[T] Events in Tonga-Fiji-Kermadec region

[S] Events in Solomon Islands

Supplementary Table 1: All events used to determine the S-wave velocity structure at the northern edge of the Pacific LLVP. Superscript T indicates events in Tonga-Fiji-Kermadec region and recorded in Alaska, along the north-south (N-S) profile. Superscript S indicates events in Solomon Islands and recorded in North America, along the west-east (W-E) profile.

Date	Lat (°)	Lon (°)	Depth (km)
2005/03/31	-18.47	176.13	12
2019/11/22	-17.72	-178.36	546
2010/03/04	-13.65	167.00	185
2011/07/29	-23.78	179.92	539
2016/05/28	-22.13	-178.17	418
2017/01/03	-19.28	176.00	17
2018/09/10	-31.92	-179.16	117
2018/09/30	-18.31	-178.11	564
2019/09/01	-20.40	-178.51	614

Supplementary Table 2: All events used to determine the P-wave velocity structure at the northern edge of the Pacific LLVP. All events are in the Tonga-Fiji-Kermadec region and recorded in Europe.

Parameters	Figure index	Profile NS-I<br>Event B (Az: 0°–10°)	Profile NS-II<br>Event B (Az: 10°–30°)	Profile NS-III<br>Event B (Dis: 90°–100°)	Profile WE<br>Event F (Dis: 100°–110°)
**δV_S/H**<br>δV_S: -30%~0<br>H: 0~100 km	Supplementary Figures 12-14	Best model with highest CC, ξ_1 and ξ_2: **δV_S = -10%, H = 50 km**			
**δV_S/H**<br>δV_S = -15%, H = 20 km	Supplementary Figure 15A	**ScS has more delays at large distance**	**There are distinct strong postcursors after ScS**	**There are distinct strong postcursors after ScS**	——
**δV_S/H**<br>δV_S = -40%, H = 10 km	Supplementary Figure 15B	**There are distinct strong postcursors after ScS**	**There are distinct strong postcursors after ScS**	**There are distinct strong postcursors after ScS**	**Sdiff postcursors are too strong with long coda wavetrain**
**Southern Boundary**<br>(western side is pushed further to the north)<br>δV_S = -10%, H = 50 km	Supplementary Figure 15C	**ScS delays are not sufficient at large distance**	——	**ScS delays are not sufficient at small azimuth**	**Sdiff postcursors arrive much earlier**
**Southern Boundary**<br>(western side is pushed further to the south)<br>δV_S = -10%, H = 50 km	Supplementary Figure 15D	**The amplitudes of ScS are much weaker at large distance**	——	**The amplitudes of ScS are much weaker at small azimuth**	——
**Northern Boundary**<br>(western side is pushed further to the south)<br>δV_S = -10%, H = 50 km	Supplementary Figure 15E	**ScS delays are not sufficient at large distance**	——	**ScS delays are not sufficient at small azimuth**	**Sdiff postcursors arrive much earlier**

**Western Boundary** (western boundary is pushed further to the east) δV_S = -10%, H = 50 km	Supplementary Figure 15F	——	——	——	**Sdiff postcursors arrive much earlier and their amplitudes are small at the azimuth of 48°-55°**
**Western Boundary** (western boundary is pushed further to the east) δV_S = -10%, H = 80 km	Supplementary Figure 15G	**ScS has more delays at large distance**	**ScS has more delays at large distance**	**ScS has more delays**	——
**Shape** (circle shape) δV_S = -10%, H = 50 km	Supplementary Figure 15H	**ScS delays are not sufficient at large distance**	——	**ScS delays are not sufficient at small azimuth**	**Sdiff postcursors arrive much earlier**
**Shape** (trapezoid shape) δV_S = -10%, H = 50 km	Supplementary Figure 15I	——	**ScS has more delays at large distance**	**ScS has more delays at large azimuth**	**Sdiff postcursors are strong at small azimuth even down to 40°**
**Shape** (irregular shape) δV_S = -10%, H = 50 km	Supplementary Figure 15J	——	——	——	**Sdiff postcursors are strong at small azimuth even down to 40°**
**Density** ($\delta\rho$ = +10%) δV_S = -10%, H = 50 km	Supplementary Figure 15K	——	——	——	——

**Density**<br>($\delta\rho$ = -10%)<br>δV_S = -10%, H = 50 km	Supplementary Figure 15L	——	——	——	——
**Density**<br>($\delta\rho$ = +20%)<br>δV_S = -10%, H = 50 km	Supplementary Figure 15M	**Slight waveform amplitude variation**	**Slight waveform amplitude variation**	**Slight waveform amplitude variation**	**Slight waveform amplitude variation**
**Non-uniform δV_S**<br>part 1: δV_S = -10%, H = 50 km<br>part 2: δV_S = -20%, H = 20 km	Supplementary Figure 15N	**ScS has more delays and strong postcursors**	**ScS has more delays and strong postcursors**	**ScS has more delays and strong postcursors**	**Sdiff postcursors are much stronger with long codas**
**Non-uniform δV_S**<br>part 1: δV_S = -10%, H = 50 km<br>part 2: δV_S = -20%, H = 20 km	Supplementary Figure 15O	**ScS delays are not sufficient at large distance**	**ScS has more delays and strong postcursors**	**ScS delays much more at large azimuth even up to 27°**	**Sdiff postcursors are much stronger with long codas**
**Non-uniform δV_S**<br>part 1: δV_S = -10%, H = 50 km<br>part 2: δV_S = -20%, H = 20 km	Supplementary Figure 15P	**ScS delays are not sufficient at large distance**	**ScS has more delays and strong postcursors**	**ScS has more delays and strong postcursors at large azimuth**	**Sdiff postcursors are strong at small azimuth even down to 40°**
**Non-uniform δV_S**<br>part 1: δV_S = -10%, H = 50 km<br>part 2: δV_S = -10%, H = 50 km	Supplementary Figure 15Q	**ScS delays are not sufficient at large distance**	**ScS has more delays at large distance**	**ScS delays much more at large azimuth even up to 22°**	**Sdiff postcursors are much stronger with long codas**

**Non-uniform δV_S**<br>δV_S = 0%~-30% (gradual),<br>varies radially,<br>H = 50 km	Supplementary Figure 15R	**ScS has more delays and strong postcursors**	**ScS has more delays and strong postcursors**	**ScS has more delays and strong postcursors**	**Sdiff postcursors are much stronger with long codas**
**Non-uniform δV_S**<br>δV_S = -5%~-15% (gradual),<br>varies radially,<br>H = 50 km	Supplementary Figure 15S	——	——	——	——
**Non-uniform δV_S**<br>δV_S = -5%~-15% (random),<br>varies radially and laterally,<br>H = 50 km	Supplementary Figure 15T	——	——	——	——
**Non-uniform δV_S**<br>Enhanced 3d tomographic model (S40RTS), mean δV_S = -10%, H = 50 km	Supplementary Figure 15U	——	——	——	——
**Non-uniform δV_S**<br>Enhanced 3d tomographic model (S40RTS), mean δV_S = -15%, H = 50 km,	Supplementary Figure 15V	**ScS has more delays and strong postcursors**	**ScS has more delays and strong postcursors**	**ScS has more delays and strong postcursors**	**Sdiff postcursors are much stronger with long codas**
**Non-uniform δV_S**<br>Enhanced 3d tomographic model (SEMUCB-WM1), mean δV_S = -15%, H = 50 km,	Supplementary Figure 15W	**ScS delays are not sufficient at large distance**	**ScS has more delays at large distance**	**ScS delays are not sufficient at small azimuth and has more delays at large azimuth**	——

**Non-uniform δVs**<br>δVs: Interpolation from $\delta t_{ScS\text{-}S}$<br>H = 50 km	Supplementary Figure 15X	——	——	——	——
**Non-uniform δVs**<br>δVs = -10%<br>H: Interpolation from $\delta t_{ScS\text{-}S}$	Supplementary Figure 15Y	——	——	——	——
**Non-uniform δVs**<br>Small discontinuous ULVZs<br>δVs = -40%, H = 20 km	Supplementary Figure 15Z	**ScS has more delays and weak amplitudes at large distance**	**ScS has more delays and weak amplitudes at large distance**	**ScS has more delays and weak amplitudes**	**Sdiff postcursors have weak amplitudes**

Supplementary Table 3: Summary of sensitivity tests for the ULVZ models. Waveform inconsistencies between synthetics and data of events B and F are described in bold while consistencies are present by lines.
